# The Relationship Between Vortical Flow and Pressure Gradient Reversal in Pulmonary Hypertension—A 4D Analysis of Cardiovascular Magnetic Resonance Flow Imaging

**DOI:** 10.1002/pul2.70350

**Published:** 2026-07-15

**Authors:** Raheeq Karim, Alexander Fyrdahl, Joao G. Ramos, Michael Melin, Patrik Sundblad, Julio Sotelo, Björn Wieslander, David Marlevi

**Affiliations:** ^1^ Unit of Clinical Physiology Karolinska University Hospital Stockholm Sweden; ^2^ Department of Molecular Medicine and Surgery Karolinska Institutet Stockholm Sweden; ^3^ Department of Cardiology, Södersjukhuset, and Department of Clinical Science and Education Karolinska Institutet, Södersjukhuset Stockholm Sweden; ^4^ Department of Medicine, Unit of Cardiology, Heart and Vascular Theme Karolinska Institute, Karolinska University Hospital Stockholm Sweden; ^5^ Division of Clinical Physiology, Department of Laboratory Medicine Karolinska Institutet Stockholm Sweden; ^6^ Departamento de Informática Universidad Técnica Federico Santa María Santiago Chile; ^7^ Department of Clinical Physiology, Research and Development Växjö Central Hospital, Region Kronoberg, Växjö, Sweden; ^8^ Institute for Medical Engineering and Science Massachusetts Institute of Technology Cambridge Massachusetts USA

**Keywords:** Hemodynamics, right heart catheterization, virtual Work Energy Relative Pressure

## Abstract

Diagnosing pulmonary hypertension (PH) requires invasive right heart catheterization (RHC). However, mean pulmonary artery pressure (mPAP) can be non‐invasively estimated using 4D analysis of cardiovascular magnetic resonance (CMR) flow imaging to assess the duration of pathological vortex flow (T_vortex_) in the main pulmonary artery (MPA). Early systolic pressure reversal has been hypothesized to drive vortex formation but has yet to be quantified in‐vivo. The virtual work‐energy relative pressure (vWERP) method enables accurate temporal characterization of intravascular pressure gradients from 4D analysis of CMR flow images. We aimed to characterize the relationship between T_vortex_ and early systolic pressure reversal in the MPA using 4D analysis of CMR flow images from patients with a range of RHC‐determined mPAP. A total of 36 patients who underwent a clinically indicated RHC for any reason were recruited for a research‐motivated CMR‐exam. Intravascular MPA pressure gradients were estimated from a stack of multidirectional 2D‐flow slices covering the pulmonary artery using vWERP. Correlations between continuous variables were assessed using linear regression analysis. There were significant but weak correlations between time to systolic pressure reversal (T_rev_) and T_vortex_ (*R* ^2^: 0.17; *p* = 0.012), pulmonary vascular resistance (PVR) (*R* ^2^: 0.29; *p* = 0.001), and mPAP (*R* ^2^: 0.27; *p* = 0.001), respectively. Multivariable analysis showed no independent correlation between T_vortex_ and T_rev_ once either PVR or mPAP were added as predictor variables. Our results suggest that early systolic pressure reversal is associated with, but is unlikely to be the sole cause of, observed vortical flow in the MPA.

Pulmonary hypertension (PH) is a serious hemodynamic condition with multiple etiologies and is defined as a mean pulmonary artery pressure (mPAP) > 20 mmHg measured using right heart catheterization (RHC) [1]. In the absence of treatment, the resulting increased afterload on the right ventricle can ultimately lead to right‐sided heart failure and death [2]. Prognosis in PH depends on etiology but is typically adverse. For example, pulmonary arterial hypertension (PAH) is associated with an overall 5‐year survival rate of approximately 60%–70% [3]. Furthermore, diagnosis is often delayed due to the gradual onset of nonspecific symptoms in combination with a high threshold for RHC‐referral [4]. In addition, RHC is associated with an approximate ~1% risk of serious complications [5]. Beyond mPAP, RHC provides key parameters such as pulmonary artery wedge pressure (PAWP) and pulmonary vascular resistance (PVR) that are essential for PH subtype classification and management [1]. Reliable and validated noninvasive methods of estimating PVR and PAWP have yet to emerge, but there are several approaches being explored [6, 7]. Currently, transthoracic echocardiography is the primary screening tool for PH in clinical routine. It utilizes the simplified Bernoulli equation to estimate systolic pulmonary arterial pressure from the tricuspid regurgitation jet velocity. However, its diagnostic accuracy is limited when compared to RHC [8], particularly for changes across repeated measurements [9]. There is thus a need for a reliable, non‐invasive method of estimating pulmonary pressures accurately.

Originally described by Reiter et al. [10], several studies have demonstrated that 4D analysis of cardiovascular magnetic resonance (CMR) images acquired using a multidirectional 2D flow approach can accurately estimate mPAP (*R* ^2^ ≥ 0.85) [10, 11, 12, 13, 14] by quantifying the duration of vortical blood flow (T_vortex_) in the main pulmonary artery (MPA). One study found 4D analysis of CMR flow images to be more sensitive than transthoracic echocardiography in diagnosing PH while maintaining specificity [13]. In addition, the use of CMR to estimate mPAP is appealing because CMR is now part of routine guidelines for PAH follow‐up to monitor right heart function [1]. While empirically described, however, the *mechanistic link* between increased mPAP and vortical flow remains largely unknown.

Potential mechanisms behind the formation of vortical flow in patients with PH have been formulated in previous studies. Notably, Reiter et al. hypothesized that the early systolic pressure reversal causes vortex formation in the MPA because the resulting relatively high systolic flow velocity against an early adverse pressure gradient is conducive to flow separation [10, 11]. However, clinical evidence of these mechanistic features remains scarce. Using Doppler‐based echocardiography, shortened pulmonary arterial acceleration time and an altered flow profile is routinely used as a sign of PH [15, 16, 17] and is typically interpreted as signifying increased PVR. However, the relationship between vortical flow in the MPA and intravascular pressure gradients remains unassessed; a gap exacerbated by Doppler‐based pressure gradient estimations typically deteriorating in accuracy when applied on complex blood flow patterns [18]. Instead, 4D analysis of CMR flow images allows for a more comprehensive assessment of intravascular pressure gradients, where recent advancements in physics‐informed image analysis now enable the derivation of pressure gradients through arbitrary vascular sections with validation performed against invasive reference data [19]. These approaches remain unexplored in the setting of PH.

Therefore, the aim of this study was to test the hypothesis that early systolic pressure reversal contributes mechanistically to blood vortices in the MPA by comparing both the observable duration of pathological flow vortices in the MPA as well as RHC findings to quantitatively estimated pressure gradients using non‐invasive 4D analysis of CMR flow images. We thereby sought to provide a better understanding of the mechanistic basis of the now established entity of vortical flow in PH.

## 1 Methods

### 1.1 Study Design and Patient Selection

In this retrospective observational study, we studied CMR exams from 36 consecutive prospectively enrolled patients who had been referred for a clinically motivated RHC for any reason, and had subsequently undergone a research‐motivated CMR exam at Karolinska University Hospital during 2017–2019 as part of a previous study [13]. Exclusion criteria were: severe claustrophobia; suspected orbital foreign body; ferromagnetic implants; pacemaker; cognitive impairment; congenital heart disease; poor image quality; irregular heart rhythm due to atrial fibrillation/flutter or excessive premature (super‐)ventricular contractions during CMR; inability to stay immobile in the supine position for the duration of the CMR scan. No contrast agents were administered. The median time between RHC and CMR was 6 days (Interquartile range, IQR:1–12 days). The clinical parameters that were documented from the RHC were mPAP, PAWP, cardiac output and PVR. All participants provided written informed consent, and the study was approved by the relevant ethics committee (Swedish Ethical Review Authority, #2017/1610‐31).

### 1.2 Right Heart Catheterization

As per clinical routine, RHC was performed using a balloon‐tipped Swan‐Ganz catheter introduced into a jugular vein. The catheter was maneuvered into the right ventricle to determine cardiac output calculated using Fick's principle. Next, the catheter was moved to the pulmonary artery to register mPAP. Finally, a balloon was inflated, and the catheter was wedged into a pulmonary artery branch to measure PAWP, which was recorded at expiration during spontaneous breathing.

### 1.3 CMR Image Acquisition

A 1.5 Tesla system was used to acquire CMR images (Siemens MAGNETOM Aera, Siemens, Erlangen, Germany). All patients underwent phase contrast flow imaging using an ECG‐gated free‐breathing multidirectional gradient‐echo 2D‐flow sagittal stack covering the MPA. Imaging parameters were: spatial resolution: 1.8 × 1.8 × 6 mm; number of averages: 3; field‐of‐view: 300–400 mm depending on patient size; repetition time (TR): 6.4 ms; echo time (TE): 4.1 ms; flip angle: 15 degrees; velocity encoding: 90 cm/s; bandwidth: 449 Hz per pixel; number of slices: 6–10, as needed to cover the MPA; acquired temporal resolution: 77 ms; reconstructed temporal resolution: 20 frames per cardiac cycle.

### 1.4 CMR Image Analysis

The vortex duration in the pulmonary artery was determined using visual analysis in a prototype software (Siemens 4D Flow, Siemens Healthcare, Erlangen, Germany) as part of a prior study, as previously described [13]. The analysis process for quantification of vortex duration has been previously described in detail, along with figures provided for additional clarity [11, 13, 20]. In summary, an imaging plane is placed using multiplanar reconstruction showing velocity vectors in the pulmonary artery an approximately sagittal view along the orientation of the right ventricular outflow tract. Next, a binary yes/no determination for vortex presence is made by the reader for each time frame of the cardiac cycle based on the presence of closed concentrical curves. The T_vortex_ is expressed as the percentage of time frames with a positive vortex finding.

**Figure 1:**
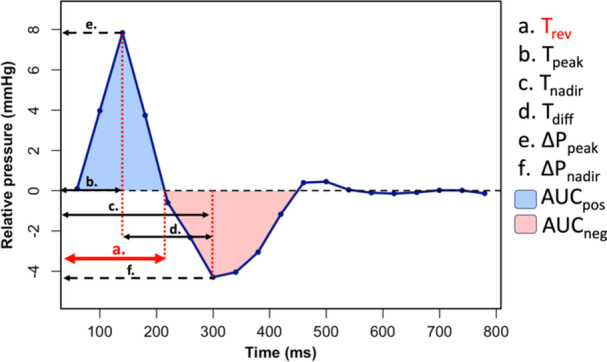
Example of a derived relative pressure trace, obtained from the 4D‐flow cardiovascular magnetic resonance imaging data. The vertical axis displays the relative pressure at a given time during the cardiac cycle. The horizontal axis displays time from the electrocardiographically triggered start of image acquisition. Abbreviations: AUC_neg_ ‐ Area under the curve on the negative side of the vertical axis. AUC_pos_ – Area under the curve on the positive side of the vertical axis; ∆P_nadir_ – nadir pressure gradient; ∆P_peak_ – peak pressure gradient; T_diff_ – time between T_peak_ and T_nadir_; T_nadir_ – time until nadir amplitude is reached; T_peak_ – time until peak amplitude is reached; T_rev_ – time until pressure gradient reversal; vWERP – virtual Work Energy Relative Pressure.

The CMR image analysis process is summarized in *Appendix 1* Figure 1. The open‐source software used for 4D flow analysis of the CMR images (4D‐Flow‐Matlab‐Toolbox) was developed using MATLAB (MATLAB version 2022a, Mathworks, Natick, Massachusetts, USA). In brief, following vessel segmentation, cross‐sectional in‐ and outlet planes were manually selected in the MPA: the inlet plane was placed immediately distal to the pulmonary valve; the outlet plane was positioned just before the pulmonary bifurcation. The virtual work‐energy relative pressure (vWERP) method [19] was then used to calculate the pressure difference between these two planes in each time frame, yielding a time‐resolved relative pressure between inlet and outlet equaling the temporal sampling of the input image data. With relative pressure data derived, we defined time to pressure reversal (T_rev_) as illustrated in Figure 1: from the start of the relative pressure curve at end diastole, determined through ECG triggering during the CMR exam, to the interpolated systolic crossing between positive and negative relative pressures. The following other parameters were also extracted for the purpose of exploratory analysis: Time to peak pressure gradient (T_peak_); time to nadir pressure gradient (T_nadir_); difference between T_peak_ and T_nadir_ (T_diff_); Peak pressure gradient amplitude (∆P_peak_); Nadir pressure gradient amplitude (∆P_nadir_); area under the curve (AUC) of the systolic positive pressure gradient (AUC_pos_) and AUC of the subsequent pressure gradient reversal (AUC_neg_). The relationship between these parameters and a pressure gradient curve are illustrated in Figure 1.

### 1.5 Inter‐ and Intraobserver Variability

The inter‐ and intra‐observer variability of the derived pressure gradient metrics was assessed in a subset of 10 patients, selected to cover a representative range of mPAP within the entire cohort. Two observers (RK and BW) independently analyzed data from 10 patients. The analysis was then repeated by the first observer (RK) after a 2‐week period to mitigate recall bias.

### 1.6 Statistical Analysis

The vWERP‐derived T_rev_ was compared with visually assessed vortex duration from a previous study in the same cohort [13], as well as invasively measured mPAP, PAWP and PVR using linear regression. Multivariable logistic regression was used to test the independent correlation of T_rev_ with T_vortex_, adding mPAP and PVR, respectively, to two separate multivariable models. User variability was reported using the intraclass correlation coefficient (ICC) and the median (IQR) of the inter‐ or intraobserver difference expressed as percent of the median cohort value for each parameter. All statistical tests were performed using the R programming language (R v.4.2.3, R core team 2022), (R Foundation for Statistical Computing, Vienna, Austria) in the software Rstudio (version 2023.09.1 + 494, Posit Software, Boston, Massachusetts, USA).

## 2 Results

### 2.1 Study Cohort

A total of 36 patients were included. The cohort consisted of 44.4% females (*N* = 16) and 55.6% males (*N* = 20). The median age of the participants was 66.5 years with an IQR of 59.0 to 75.3 years. Cohort characteristics are presented in Table 1. Summarized inter‐ and intraobserver variability results are presented in Table 2.

**Table 1:** Cohort characteristics.

Patient characteristics	All patients (*n* = 36)
Female, *n* (%)	16 (44)
Age, years (mean ± standard deviation)	63.8 ± 19.4
BMI, kg/m^2^ (mean ± standard deviation)	26.5 ± 6.1
Subgroups	
Heart transplanted, *n* (%)	10 (28)
Suspected or confirmed pulmonary hypertension, *n* (%)	28 (78)
RHC diagnosis	
Precapillary or combined pulmonary hypertension, *n* (%)	17 (47)
Isolated postcapillary pulmonary hypertension, *n* (%)	7 (19)
RHC parameters	
mPAP, mmHg, median (IQR)	28.5 (19.0–36.0)
PVR, WU, median (IQR)	3.4 (1.6–4.7)
PAWP, mmHg, median (IQR)	11.0 (7.0–16.5)
CMR parameters	
T_vortex_, % of the cardiac cycle duration, median (IQR)	27.5 (10.0–35.0)
RVEDVI, ml/m^2^, median (IQR)	98 (69–138)
RVEF, %, median (IQR)	43 (28–51)

Abbreviations: BMI, body mass index; IQR, inter‐quartile range; mPAP, mean pulmonary artery pressure; PAWP, pulmonary artery wedge pressure; PVR, pulmonary vascular resistance; RHC, right heart catheterization; RVEDVI, right ventricle end diastolic volume index; RVEF, right ventricle ejection fraction; T_vortex_, duration of vortical blood flow in the pulmonary artery as percent of one heart cycle; WU, woods units.

**Table 2:** Inter‐ and intraobserver variability.

Parameter	Interobserver		Intraobserver	
	ICC	Median (IQR) difference in % of cohort median	ICC	Median (IQR) difference in % of cohort median
T_rev_	0.72	7 (4–10)	0.74	3 (1–11)
T_peak_	0.76	0 (0–0)	0.80	0 (0–0)
T_nadir_	0.91	0 (0–8)	0.79	0 (0–7)
T_diff_	0.85	7 (0–16)	0.77	0 (0–10)
∆P_peak_	0.59	26 (18–35)	0.73	37 (15–89)
∆P_nadir_	0.50	31 (27–47)	0.86	23 (12–62)
AUC_pos_	0.59	31 (20–36)	0.59	24 (16–91)
AUC_neg_	0.49	33 (16–60)	0.86	27 (14–63)

Abbreviations: AUC_neg_, area under the curve on the negative side of the vertical axis; AUC_pos_, area under the curve on the positive side of the vertical axis; ICC, intraclass correlation coefficient; ∆P_nadir_, nadir pressure gradient; ∆P_peak_, peak pressure gradient; T_diff_, time between T_peak_ and T_nadir_; T_nadir_, time until nadir amplitude is reached; T_peak_, time until peak amplitude is reached; T_rev_, time until pressure gradient reversal.

### 2.2 Linear Regression Analysis

**Figure 2:**
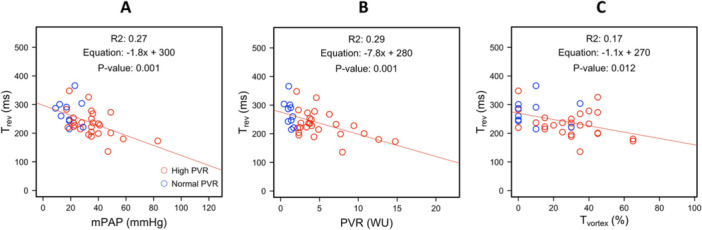
Scatter plots illustrating the relationships between T_rev_ and key hemodynamic parameters. (A) T_rev_ versus mPAP. (B) T_rev_ versus PVR. (C) T_rev_ versus T_vortex_. Each plot includes a linear regression line with corresponding *R* ^2^ and *p*‐values. Data points are color‐coded by PVR status: red circles indicate elevated PVR, and blue circles indicate normal PVR. Abbreviations: mPAP – mean pulmonary artery pressure; PAWP – pulmonary artery wedge pressure; PVR – pulmonary vascular resistance; T_rev_ – time until pressure gradient reversal; T_vortex_ – duration of vortical blood flow in the pulmonary artery as a percent of one heart cycle; WU – Wood units.

Among the variables derived from the time‐resolved relative pressure trace, T_rev_ and AUC_pos_ each had statistically significant linear correlations with T_vortex_, mPAP and PVR, respectively. The strongest correlation for T_rev_ was the inverse relationship with PVR (*R* ^2^: 0.29; *p* = 0.001). Scatter plots between T_rev_ and T_vortex_, mPAP, and PVR, respectively, are shown in Figure 2. Linear regression analysis between relative pressure parameter and each of the RHC‐measures, in addition to T_vortex_, are also shown in Table 3.

**Table 3:** Univariable regression analysis.

	∆P_peak_ mmHg	∆P_nadir_ mmHg	T_peak_ ms	T_nadir_ ms	T_diff_ ms	T_rev_ ms	AUC_pos_ s*mmHg	AUC_neg_ s*mmHg
T_vortex_								
*R* ^2^	0.12	0.03	0.02	0.01	0.02	0.17	0.28	0.01
Coefficient	−0.026	0.007	−0.26	0.35	0.61	−1.10	−0.004	0.001
*p*	**0.040**	0.30	0.36	0.63	0.38	**0.012**	**0.001**	0.59
mPAP								
*R* ^2^	0.11	0.03	0.06	0.01	0	0.27	0.26	0
Coefficient	−0.032	0.008	−0.53	−0.47	0.06	−1.75	−0.004	0.000
*p*	**0.043**	0.31	0.14	0.60	0.95	**0.001**	**0.002**	0.69
PVR								
*R* ^2^	0.11	0.02	0.06	0.01	0	0.29	0.2	0
Coefficient	−0.136	0.026	−2.17	−2.28	−0.11	−7.75	−0.017	0.000
*p*	**0.045**	0.47	0.15	0.55	0.98	**0.001**	**0.006**	0.93
PAWP								
*R* ^2^	0.01	0.02	0.01	0	0	0.01	0.04	0.05
Coefficient	−0.016	0.015	−0.47	−0.42	0.05	−0.55	−0.004	0.004
*p*	0.67	0.42	0.58	0.84	0.98	0.68	0.22	0.20

*Note:* Statistically significant *p*‐values (< 0.05) are marked in bold. Coefficient represents the slope in a linear regression analysis.

Abbreviations: AUC_neg_, area under the curve on the negative side of the vertical axis. AUC_pos_, area under the curve on the positive side of the vertical axis; mPAP, mean pulmonary artery pressure; PAWP, pulmonary artery wedge pressure; ∆P_nadir_, nadir pressure gradient; ∆P_peak_, peak pressure gradient; PVR, pulmonary vascular resistance; T_diff_, time between T_peak_ and T_nadir_; T_nadir_, time until nadir amplitude is reached; T_peak_, time until peak amplitude is reached; T_rev_, time until pressure gradient reversal; T_vortex_, duration of vortical blood flow in the pulmonary artery as percent of one heart cycle.

### 2.3 Multivariable Analysis

Multivariable analysis was performed using T_vortex_ as the outcome variable and T_rev_ as the predictor variable, with PVR and mPAP beind added as a co‐variable one at a time, see Table 4. The multivariable analysis revealed that the association between T_vortex_ and T_rev_ was no longer significant once either PVR or mPAP was added to the model.

**Table 4:** Multivariable regression analysis.

Outcome variable	Predictor variables	*p*	Coefficient	Model *R* ^2^
T_vortex_	T_rev_	0.98	0.001 (%/ms)	0.59
	PVR	**< 0.001**	4.17 (%/WU)	
T_vortex_	T_rev_	0.34	0.03 (%/ms)	0.83
	mPAP	**< 0.001**	1.21 (%/mmHg)	

*Note:* Statistically significant *p*‐values (< 0.05) are marked in bold.

Abbreviations: PVR, pulmonary vascular resistance; T_rev_, time until pressure gradient reversal; T_vortex_, duration of vortical blood flow in the pulmonary artery as percent of one heart cycle; WU, Wood units.

### 2.4 Subgroup Analysis

The patient cohort was divided into two groups based on whether the PVR was normal (≤ 2 WU) or abnormally elevated (> 2 WU), suggesting a precapillary component in PH etiology. These groups were then analyzed using linear regression, both including and excluding heart‐transplanted patients (see Appendix 3 for scatterplots). A significant correlation was found between T_rev_ and both PVR and mPAP in the elevated PVR groups (including and excluding heart‐transplanted patients), with *R* ^2^ values ranging from 0.22 to 0.25 (*p* < 0.05; *n* = 26 when including, or *n* = 22 when excluding heart‐transplanted patients). No significant correlations were observed in the normal PVR groups (*n* = 10, or *n* = 4 when excluding heart‐transplanted patients), nor in any group with respect to T_vortex_. Results from the subgroup analysis are presented in Table 5.

**Table 5:** Linear regression subgroup analysis.

Predictor variable	Outcome variable	*p*	Coefficient	Model *R* ^2^
PVR ≤ 2 (*n* = 10)				
T_rev_	mPAP	0.87	−0.42	< 0.01
	PVR	0.12	−66.8	0.27
	T_vortex_	1	0	< 0.01
PVR > 2 (*n* = 26)				
T_rev_	mPAP	**0.012**	−1.56	0.23
	PVR	**0.012**	−6.53	0.23
	T_vortex_	0.071	−0.95	0.13

*Note:* Statistically significant *p*‐values (< 0.05) are marked in bold.

Abbreviations: mPAP, mean pulmonary artery pressure; PVR, pulmonary vascular resistance; T_rev_, time until pressure gradient reversal; T_vortex_, duration of vortical blood flow in the pulmonary artery as percent of one heart cycle.

### 2.5 Exploratory Analyses

There was a weak but significant inverse correlation between AUC_pos_ and T_vortex_ (*R* ^2^ = 0.28, *p* = 0.001), mPAP (*R* ^2^ = 0.26, *p* = 0.006) and PVR (*R* ^2^ = 0.20, *p* = 0.006), respectively, as well as between ∆P_peak_ and T_vortex_ (*R* ^2^ = 0.12, *p* = 0.04), mPAP (*R* ^2^ = 0.11, *p* = 0.043) and PVR (*R* ^2^ = 0.11, *p* = 0.045). For the remaining quantitative pressure gradient variables (illustrated in Figure 1), there was no correlation with T_vortex_ or any RHC‐derived variable, including PAWP (see Table 3).

### 2.6 Inter‐ and Intraobserver Variability

Inter‐ and intraobserver variability data are summarized in Table 2. The interobserver ICC for time domain variables (T_rev_, T_peak_, T_nadir_ and T_diff_) ranged between 0.72 and 0.91. The interobserver ICC for variables involving relative pressure amplitudes (∆P_peak_, ∆P_nadir_, AUC_pos_, AUC_neg_) ranged between 0.49 and 0.59, indicating lower reproducibility than that of the studied time domain variables (see Figure 1 for illustrated definitions of the above‐mentioned variables). The median (IQR) interobserver difference in T_rev_ was 17 (9–25) ms, corresponding to 7 (4–10) % of the cohort median T_rev_ of 237 ms. The median (IQR) intraobserver variability was 8 (2–27) ms or 4 (1–11) % of the median T_rev_. Bland‐Altmann plots showing detailed inter‐ and intraobserver variability results are provided in Appendix 2.

## 3 Discussion

The aim of this study was to test the previously posed hypothesis that early systolic pressure reversal contributes to the emergence of pathological vortical flow in the MPA in the setting PH [10]. The major finding of our work was that the time to systolic pressure reversal was weakly correlated with the duration of vortical blood flow in the MPA and that this correlation was not independent of either PVR or mPAP in a multivariable analysis. These observations did not support our hypothesis but instead suggest that early systolic pressure reversal is unlikely to be a primary driver of vortex formation. Our results constitute a step toward a better understanding of the changed hemodynamic circumstances that cause MPA vortices in PH through the contribution of empirical data.

In our analysis, we only observed statistically significant correlations between T_rev_ and mPAP, T_vortex_ and PVR, respectively, in patients with high PVR, suggesting that early systolic pressure reversal might primarily occur in the presence of *precapillary* PH. In contrast to this, however, early systolic pressure reversal has been indirectly observed in both pre‐ and postcapillary PH by echocardiography through measurements of shortened pulmonary artery acceleration time. Shortened pulmonary artery acceleration time has long been used clinically as a marker for elevated PVR and mPAP [15, 17, 21], and empirical relationships have even been proposed to roughly estimate mPAP based on pulmonary artery acceleration time [15]. Pulmonary artery acceleration time can be thought of as analogous to T_rev_ since flow velocity theoretically accelerates until the pressure gradient reverses, although vessel wall elasticity and blood viscosity may temporally decouple peak flow velocity and pressure reversal. The routine clinical echocardiographic estimation of pulmonary arterial pressure based on tricuspid or pulmonary regurgitation velocity similarly relies on underlying assumptions, which become violated when extending into multidimensional flows with substantial bias reported against reference pressure data [8, 18, 22, 23, 24]. In comparison, use of 4D analysis of CMR flow images and the vWERP method omits any assumption on the underlying flow, allowing for more accurate estimation of pressure gradients in complex flow environments [19, 25].

In addition to the assessment of intravascular vortical flows, increased pulmonary artery pulse wave velocity has also been associated with PH [26]. Herein, early systolic pressure reversal could be expected to be associated with increased pulse wave velocity, where distal wave reflections return earlier with a more rapidly propagating forward pressure wave. With pulse wave velocity typically modulated by vessel compliance, these associated changes could then be theoretically argued to either be linked to vessel distensions observed in postcapillary PH, or increased vessel stiffness occurring in precapillary or combined PH. Along the same lines, it has been previously suggested [27] that early systolic pressure reversal is primarily related to pulmonary vascular impedance, a property analogous to PVR that considers the pulsatility of flow. The concept of vascular impedance may be useful in this context as it captures both vascular resistance as well as phenomena affecting vascular capacitance and thereby reactance, such as vessel dilatation, stiffness and distension. The normal pulmonary vascular environment of low resistance and high capacitance is physiologically adapted to conserve kinetic energy by continued forward motion of blood and distension of elastic vascular walls. However, in the setting of high impedance, i.e., high resistance and low capacitance, early systolic pressure reversal may result from rapid pulse wave velocity and high resistance to forward flow, while kinetic energy conservation may increasingly occur through vortical flow. Indeed, in a previous study using *in silico* modeling of flow in the pulmonary artery based on in vivo data, increasing right ventricular afterload in isolation did not cause vortex formation, but altering the vessel shape did [28]. This finding by Sabry and colleagues is compatible with the notion that vessel dilatation and stiffening may be conducive to vortex formation, potentially through flow separation along the posterior vessel wall as suggested by Reiter et al. [10]. Early systolic pressure reversal is likely to follow from the decreased capacitance in this environment, rather than acting as a primary driver of the formation of vortical flow.

As postulated by Reiter [10] and Helderman [29], vortex formation in the MPA has been hypothesized to be linked to early systolic pressure reversal, following from a combination of vessel dilatation and stiffening, contributing to intravascular flow separation appearing along a posterior boundary layer of the MPA. The presence of a thickened boundary layer – in turn following an increased pressure state – has then been thought to set the condition for vortical blood flow. Our results indeed show a weak but observable association between T_vortex_ and T_rev_. Nevertheless, the weakness of the correlation, along with along with the loss of statistical significance in the multivariable regression analysis suggests the presence of other primary causes of MPA vortex formation other than early systolic pressure reversal.

In secondary exploratory analyses, we found that parameters incorporating intravascular pressure gradient amplitude (∆P_peak_ and AUC_pos_) correlated significantly with T_vortex_ as well as with mPAP and PVR. However, we refrain from drawing conclusions from this finding because the interobserver variability analysis indicated limited reproducibility for these parameters, suggesting that they may be materially influenced by differences across observers in vessel segmentation or placement of inlet and outlet planes.

Interobserver variability of the manual assessment of vortex duration has been previously shown to be excellent in the study cohort (vortex duration difference 95% CI: 0%–3%, corresponding to 0–1.9 mmHg; intraclass correlation coefficient 0.93) [13]. Similarly, Reiter et al. also reported excellent interobserver agreement (vortex duration difference 95% CI: 2.7%–3.4%, corresponding to 1.7–2.1 mmHg; intraclass correlation coefficient 0.98) in a separate cohort [11]. Nevertheless, the manual nature of the method comes with inherent user‐dependency. While efforts to automate vortex detection are underway [12], these remain to be tested in larger datasets as well as be made public to the larger research community.

The sample size was limited, although likely sufficient to achieve the aim of the study in characterizing the approximate strength of the relationship between early systolic pressure reversal and vortex in the MPA. The limited reconstructed temporal resolution of 20 frames per cardiac cycle may have limited the precise determination of T_rev_ as well as of vortex onset and termination. A future study with improved temporal resolution may further elucidate the statistical association between T_rev_ and T_vortex_ by removing this limitation. The risk of systematic bias in the observed association may have been partially mitigated as T_rev_ and T_vortex_ were both derived from the same temporally resolved dataset. Moreover, a similar temporal resolution has been used in previous studies when correlating T_vortex_ and RHC‐derived mPAP [11], where strong associations were observed and validated against invasive measurements.

Although mPAP and T_vortex_ have been empirically linked, the mechanistic assessment of their interlinked nature has been a limiting factor in understanding the appropriate clinical role of T_vortex_ as an imaging biomarker of mPAP. Our results do not support that early systolic pressure reversal is a primary cause of vortex formation, but instead implies that other mechanisms are required to fully explain vortex formation in the setting of PH. Hence, our study contributes to the body of evidence needed for clinical translation, while still pointing out remaining unknowns. Further studies are needed before T_vortex_ is used clinically, particularly for the purpose of monitoring short‐term changes in mPAP.

Further elucidation of the mechanism underlying vortex formation could potentially be achieved through future prospective studies with controlled hemodynamic interventions. For instance, an experimental intervention to acutely alter mPAP during simultaneous RHC and 4D flow CMR could help better isolate the direct effects between pressure reversal and T_vortex_. Such an approach may clarify whether T_vortex_ and T_rev_ are primarily affected by chronic vascular remodeling or whether they also respond to acute changes in pulmonary pressure in the setting of PH. In addition, vWERP‐determined T_rev_ could conceivably be explored as a novel biomarker in PH, although that would require sufficiently powered prospective studies to confirm robustness and clinical utility.

In conclusion, we observed a weak correlation between time to systolic pressure reversal and duration of vortical flow in the MPA using 4D analysis of multidirectional 2D CMR flow images. However, as this association was not independent of either mPAP or PVR, our data did not support the hypothesis that early systolic pressure reversal is the primary driver of vortex formation in the setting of pulmonary hypertension.

## Author Contributions

Conception and study design: Björn Wieslander and David Marlevi; Data acquisition: Björn Wieslander, Joao G. Ramos, Michael Melin, Patrik Sundblad, Alexander Fyrdahl; Data curation; Björn Wieslander, Joao G. Ramos, Raheeq Karim and Alexander Fyrdahl; Software: David Marlevi and Julio Sotelo; Data analysis: Raheeq Karim, Björn Wieslander; Results interpretation: Raheeq Karim, Björn Wieslander and David Marlevi. Manuscript drafting: Raheeq Karim, Björn Wieslander; Manuscript critical revision: Raheeq Karim, Alexander Fyrdahl, Joao G. Ramos, Michael Melin, Patrik Sundblad, Julio Sotelo, Björn Wieslander and David Marlevi. Funding acquisition: Björn Wieslander and David Marlevi. All authors read and approved the final manuscript.

## Ethics Statement

This retrospective observational study was conducted in accordance with the declaration of Helsinki. All participants provided written informed consent upon inclusion and the study was approved by the relevant ethics committee (Swedish Ethical Review Authority, #2017/1610‐31).

## Conflicts of Interest

The authors declare no conflicts of interest.

## Supporting information

Supporting File

## Data Availability

The data that support the findings of this study are available on request from the corresponding author. The data are not publicly available due to privacy or ethical restrictions.
