# Supplementary material for: The Relationship Between Vortical Flow and Pressure Gradient Reversal in Pulmonary Hypertension—A 4D Analysis of Cardiovascular Magnetic Resonance Flow Imaging
Source: Pulm Circ. 2026 Jul 15;16(3):e70350. doi: 10.1002/pul2.70350 (PMC13404771; doi:10.1002/pul2.70350)
Supplement: Supplementary file 1 — Supporting File [file PUL2-16-e70350-s001.docx]

**Appendix 1 – Overview of the image analysis process**


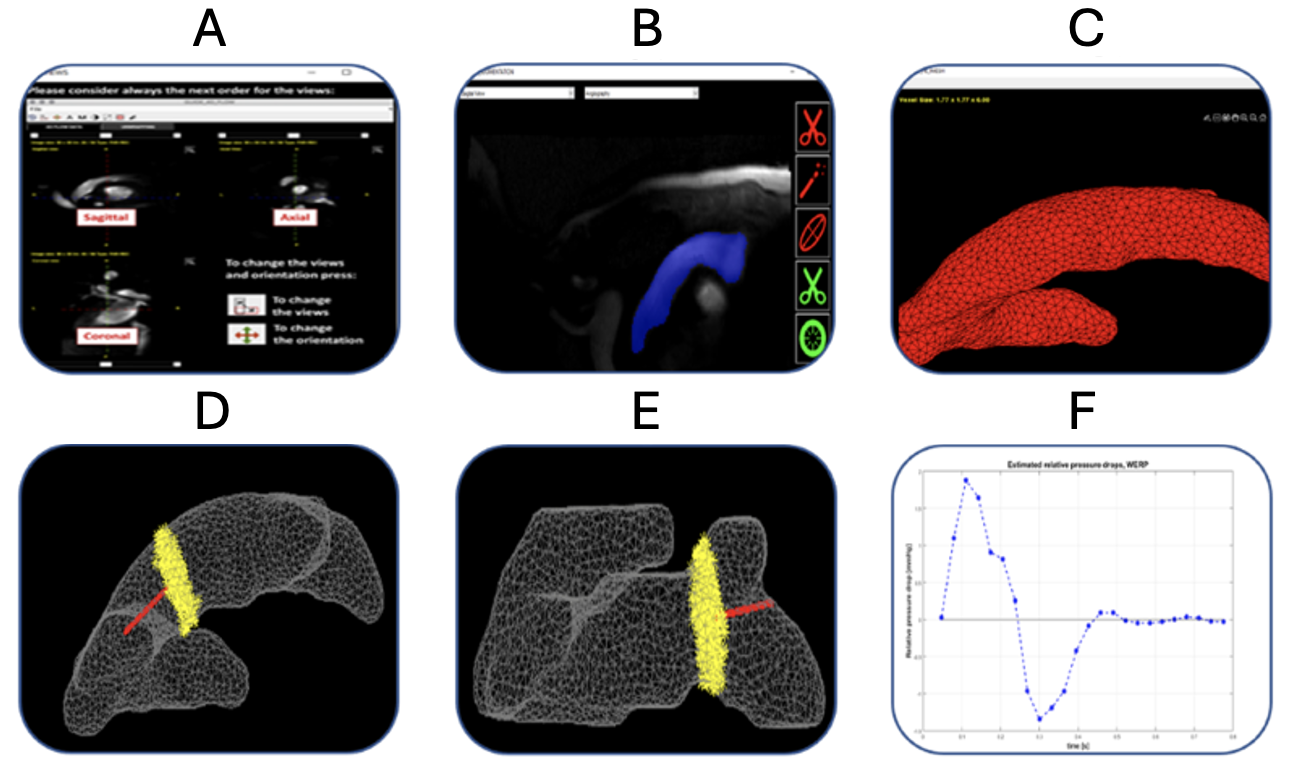


***Appendix 1, supplemental figure 1. Overview of the image analysis process.*** *Once images were loaded into the 4D Flow APP*^28,29^ *(Panel A), the pulmonary artery is semi-automatically segmented (Panel B). Next, a three-dimensional mesh was generated of the pulmonary artery (panel C), in which an inlet plane (panel D) was placed just distally to the pulmonary valve and an outlet plane (panel E) was placed at the pulmonary artery bifurcation. Finally, the pressure gradient was calculated in each time frame using the virtual work-energy relative pressure (vWERP)-function, generating a pressure drop curve covering one heart cycle (panel F).*

**Appendix 2 – Detailed inter- and intraobserver variability data**

| 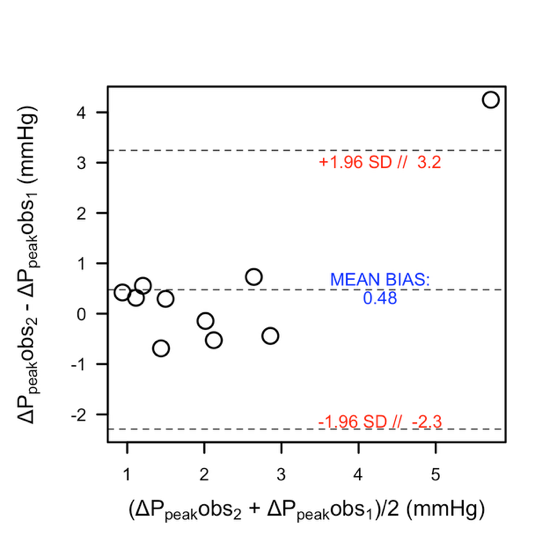 | 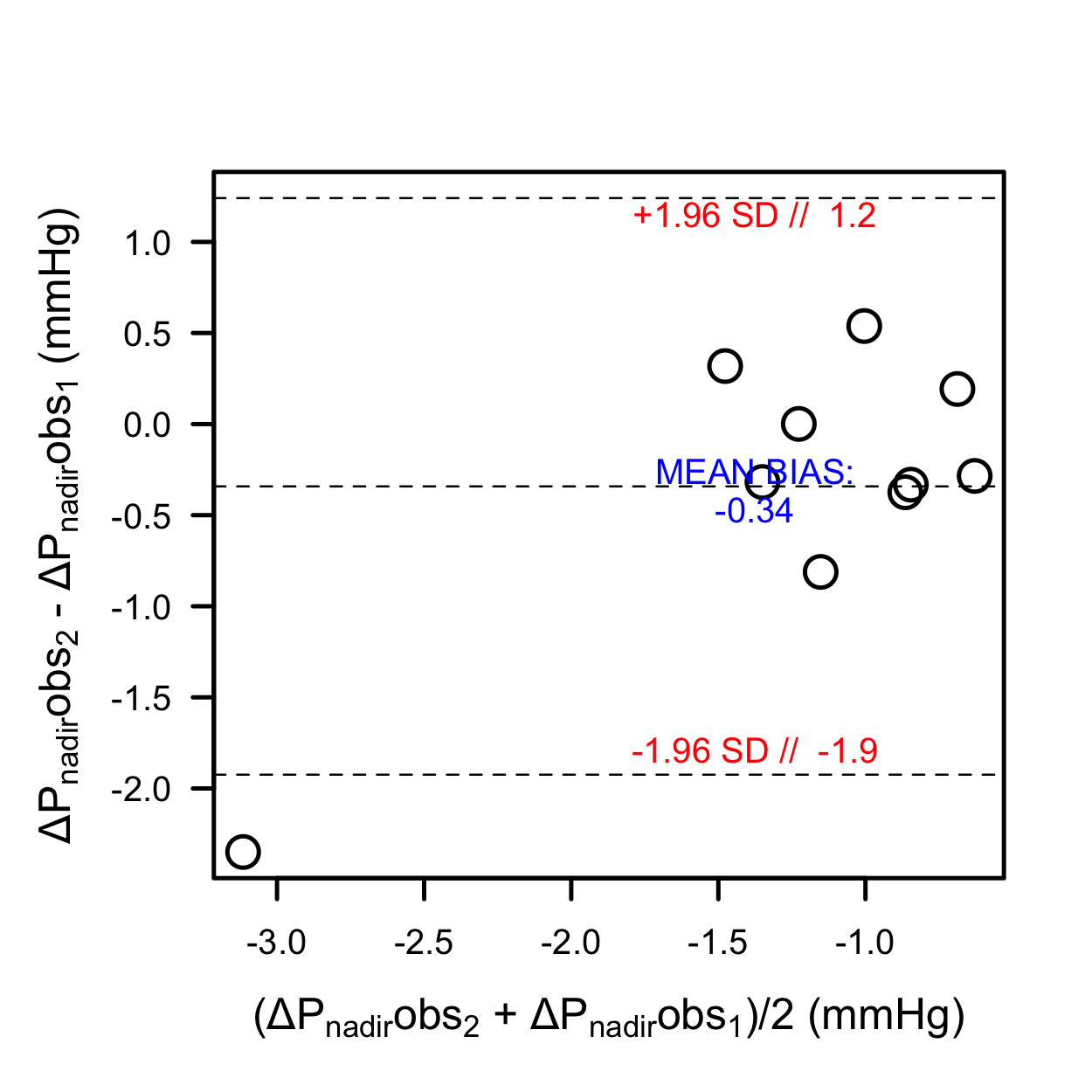 |
| --- | --- |
| 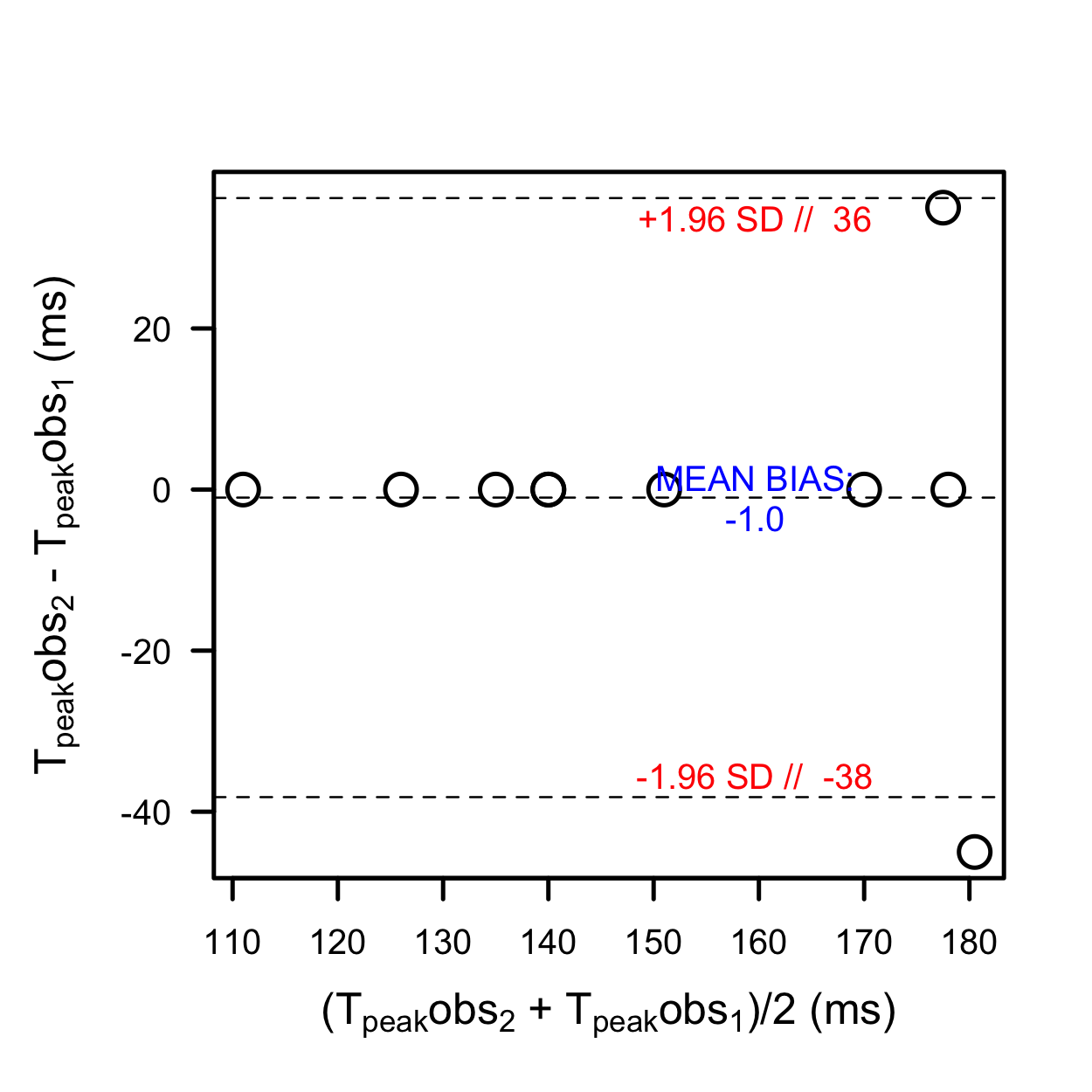 | 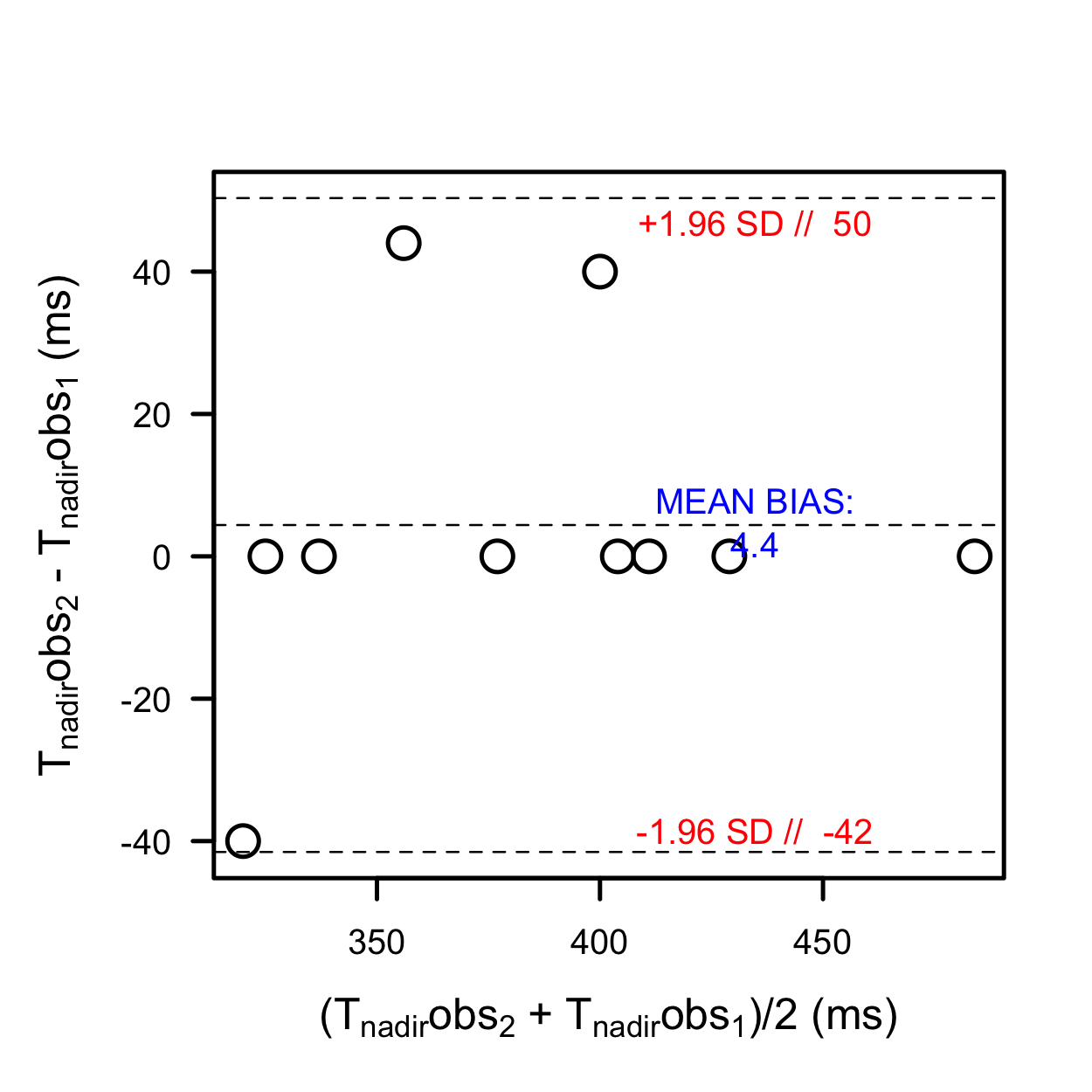 |
| 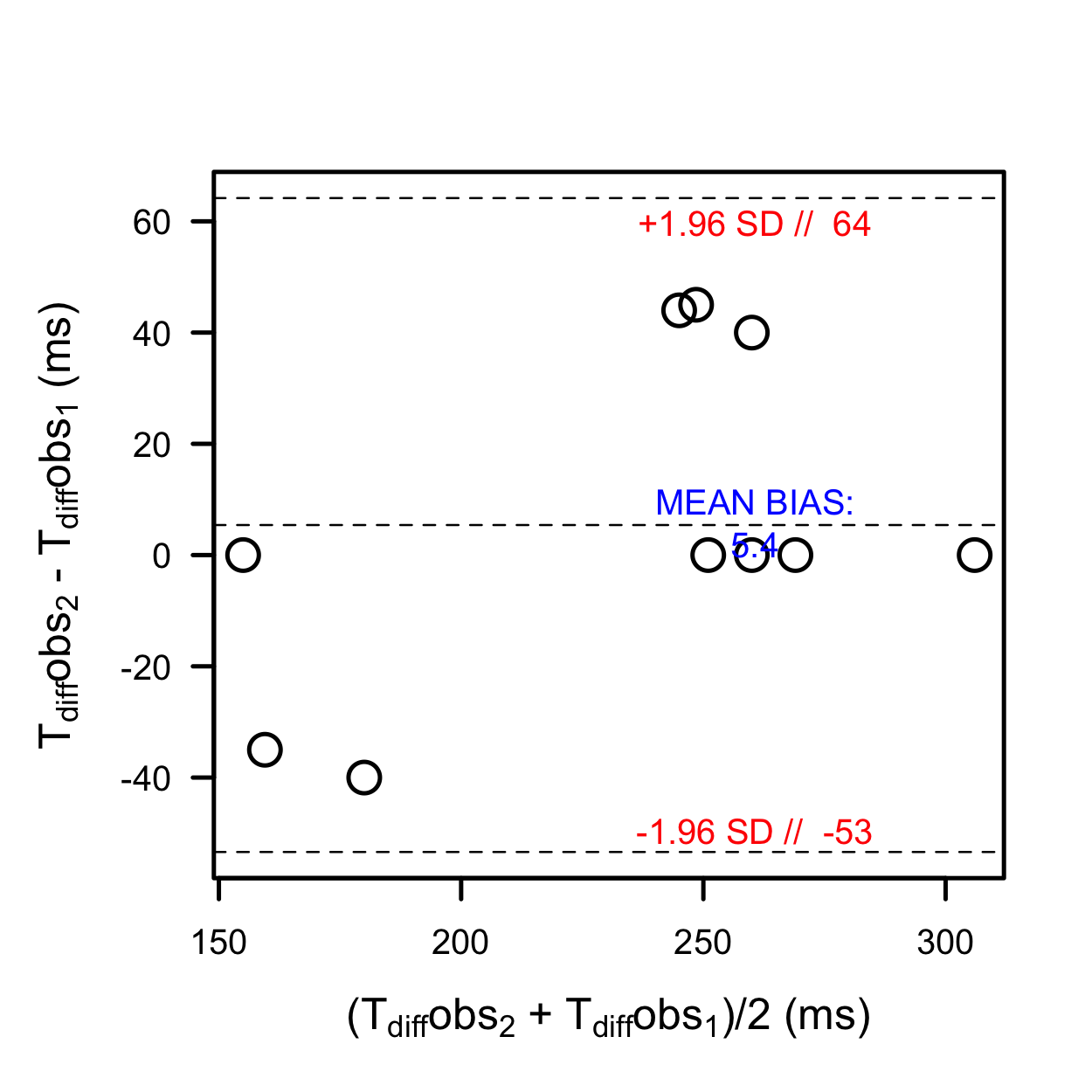 | 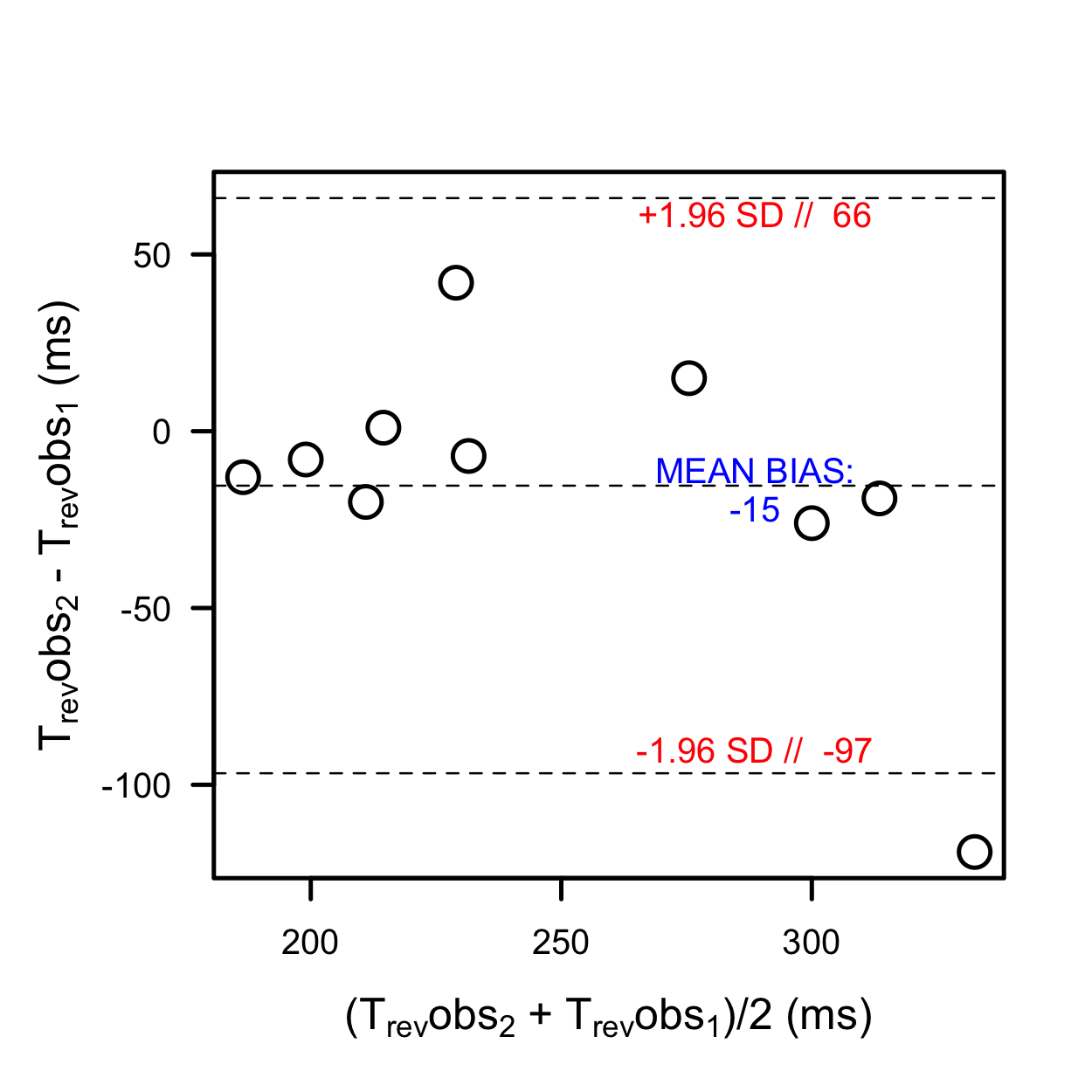 |
| 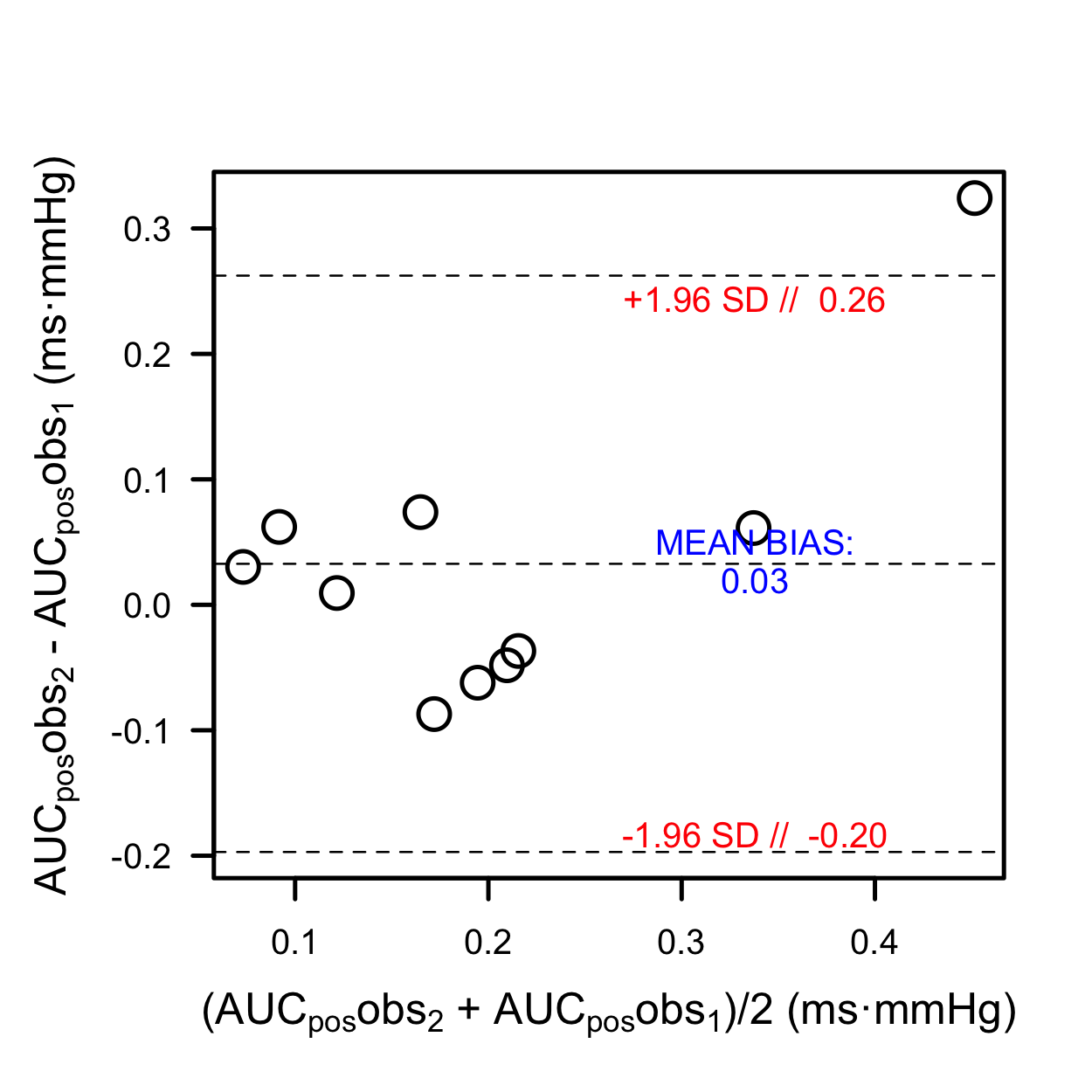 | 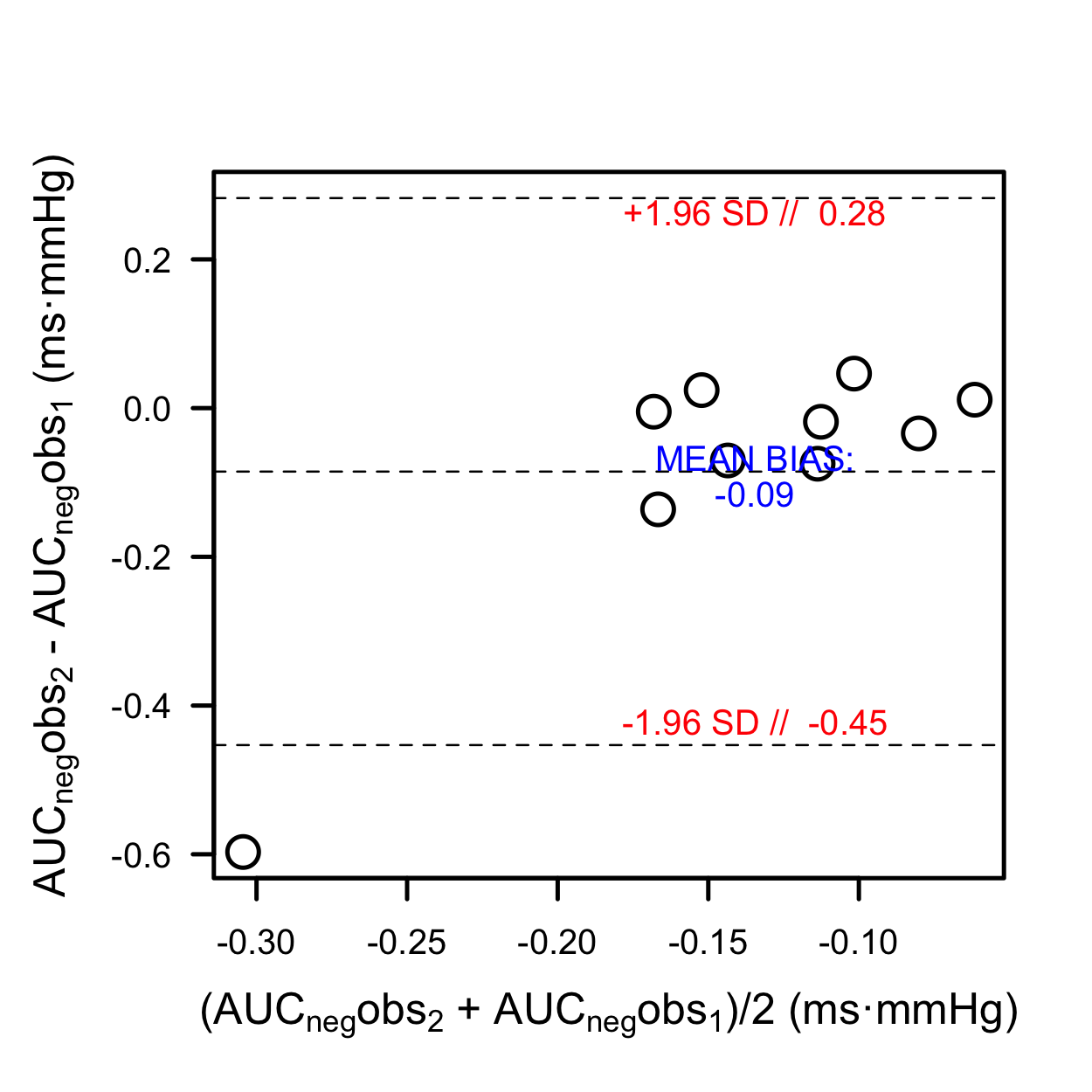 |

***Appendix 2, supplemental figure 1.****Inter-observer variability assessment using Bland-Altman plots which depict bias (mean difference, solid line) and 95% limits of agreement (dashed lines) for all variables derived from vWERP.*

*Abbreviations: Obs – observer; T_diff_ – time between T_peak_ and T_nadir_; T_nadir_ – time until nadir amplitude is reached; T_peak_ – time until peak amplitude is reached; T_rev_ – time until pressure gradient reversal.*

**Appendix 2, supplemental figure 2 – intraobserver variability**

| 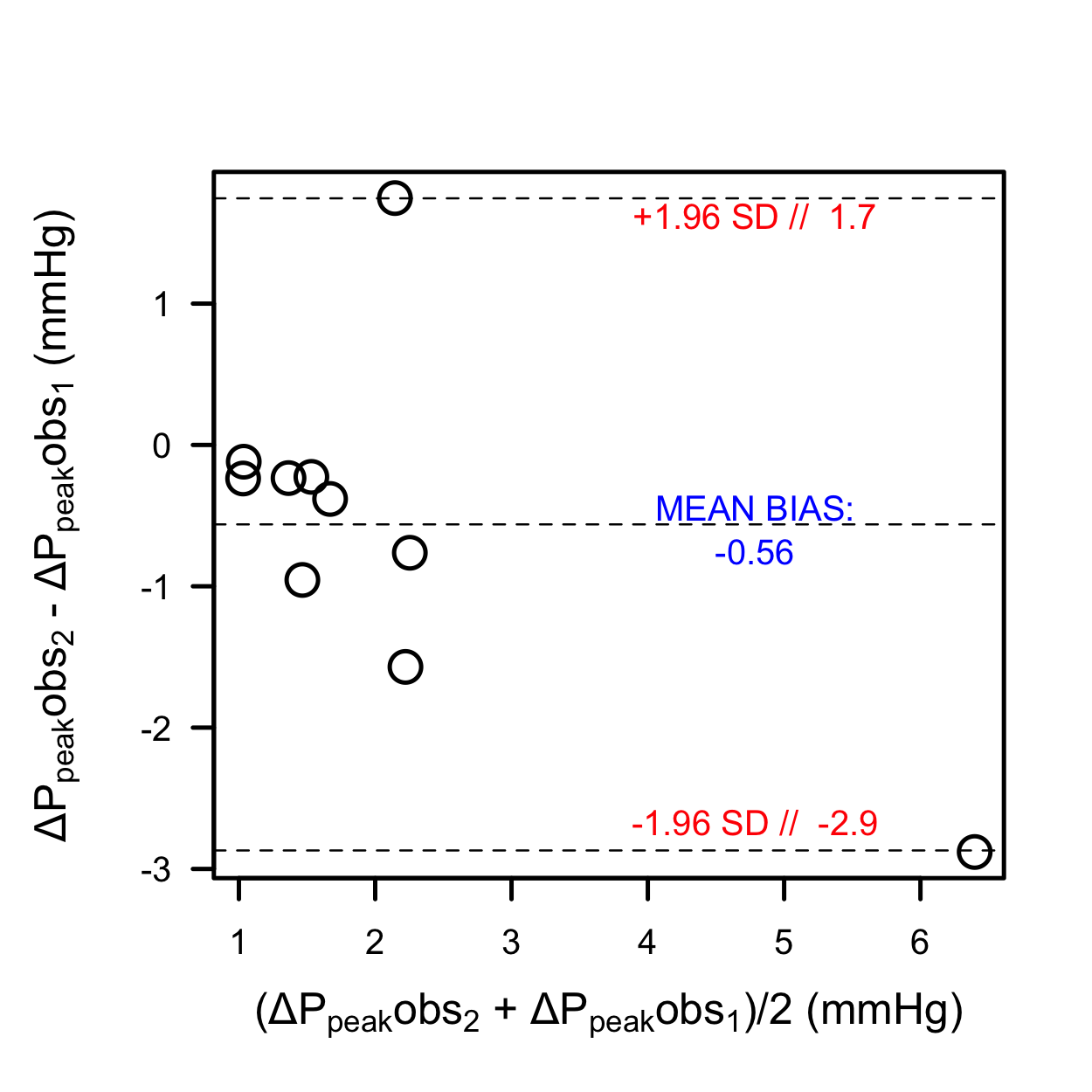 | 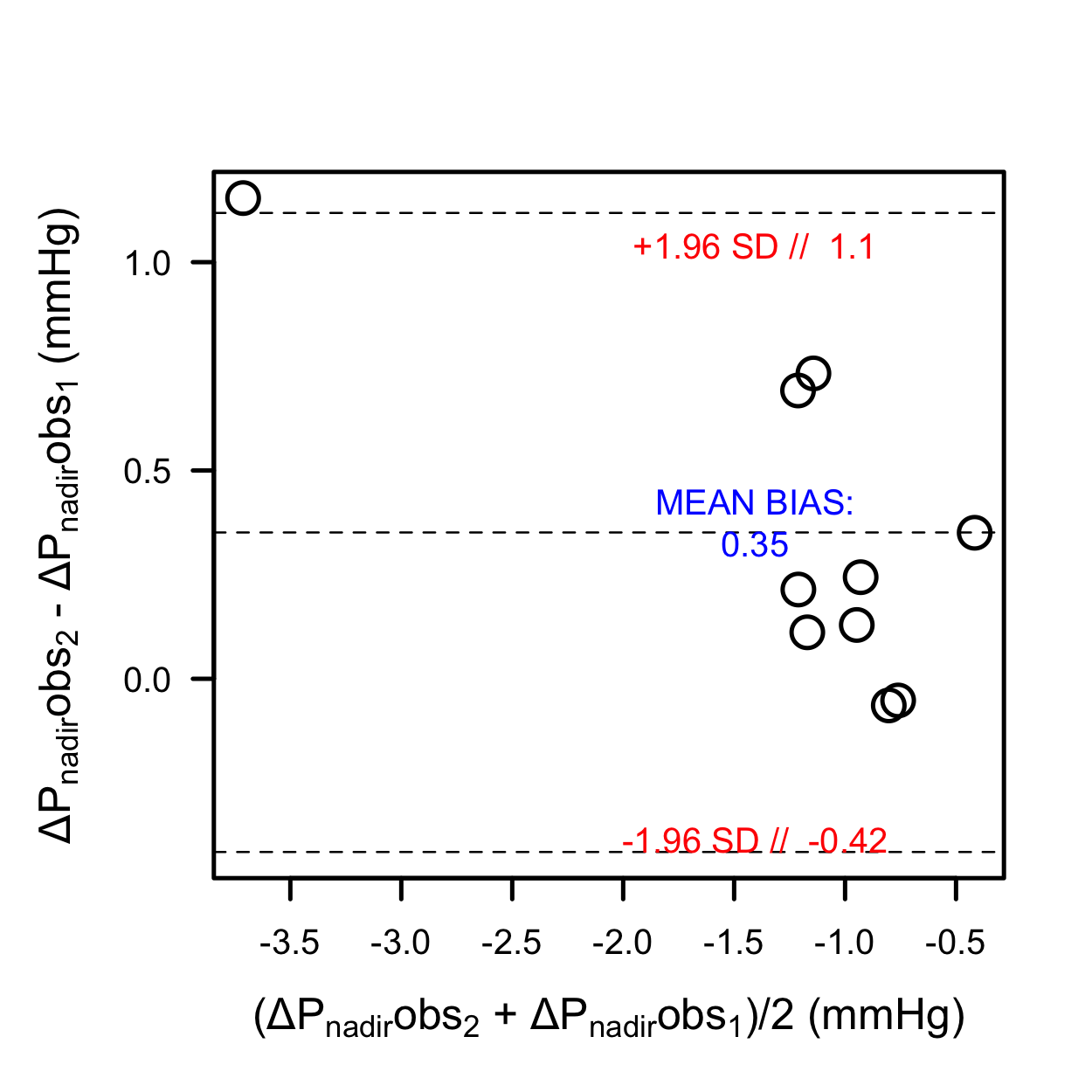 |
| --- | --- |
| 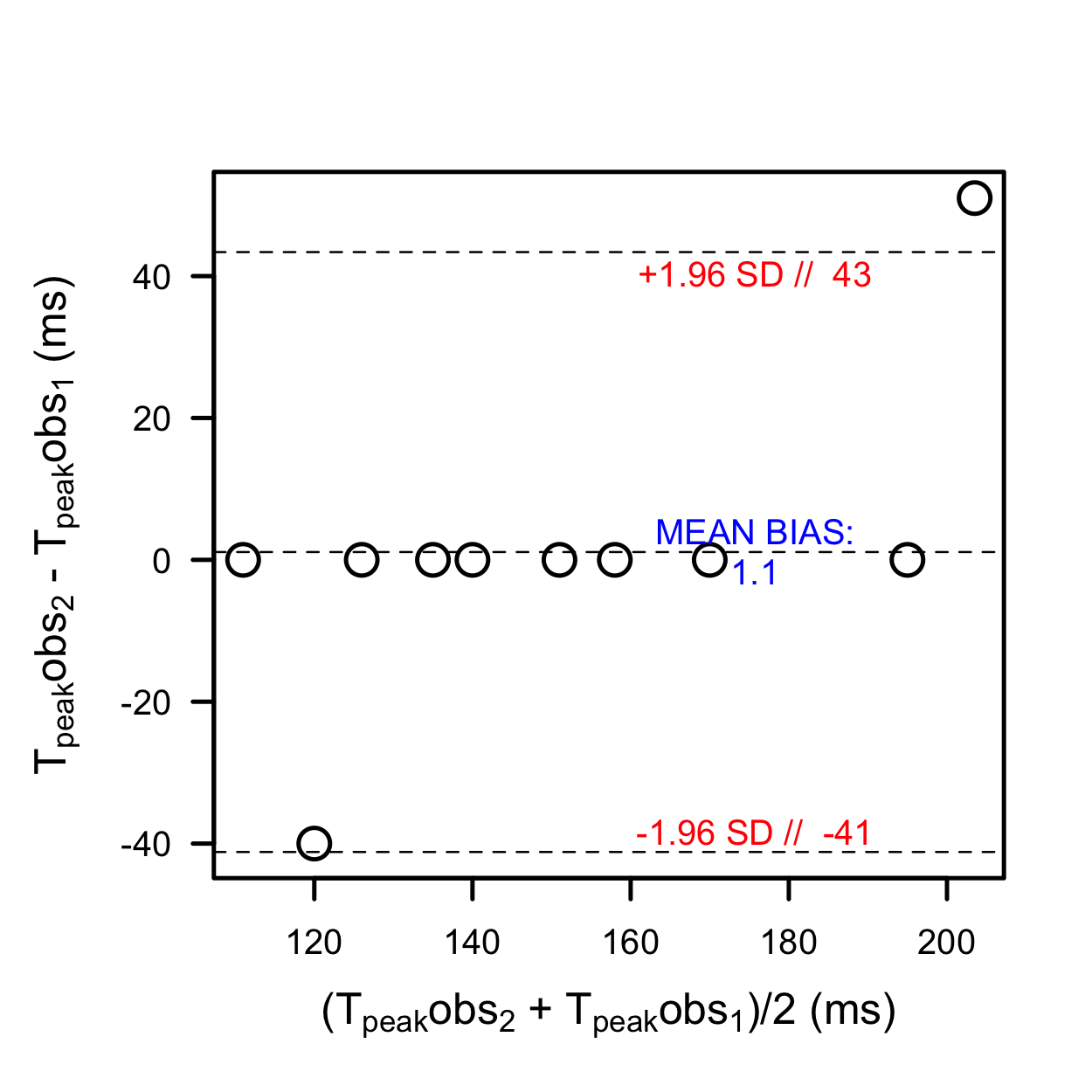 | 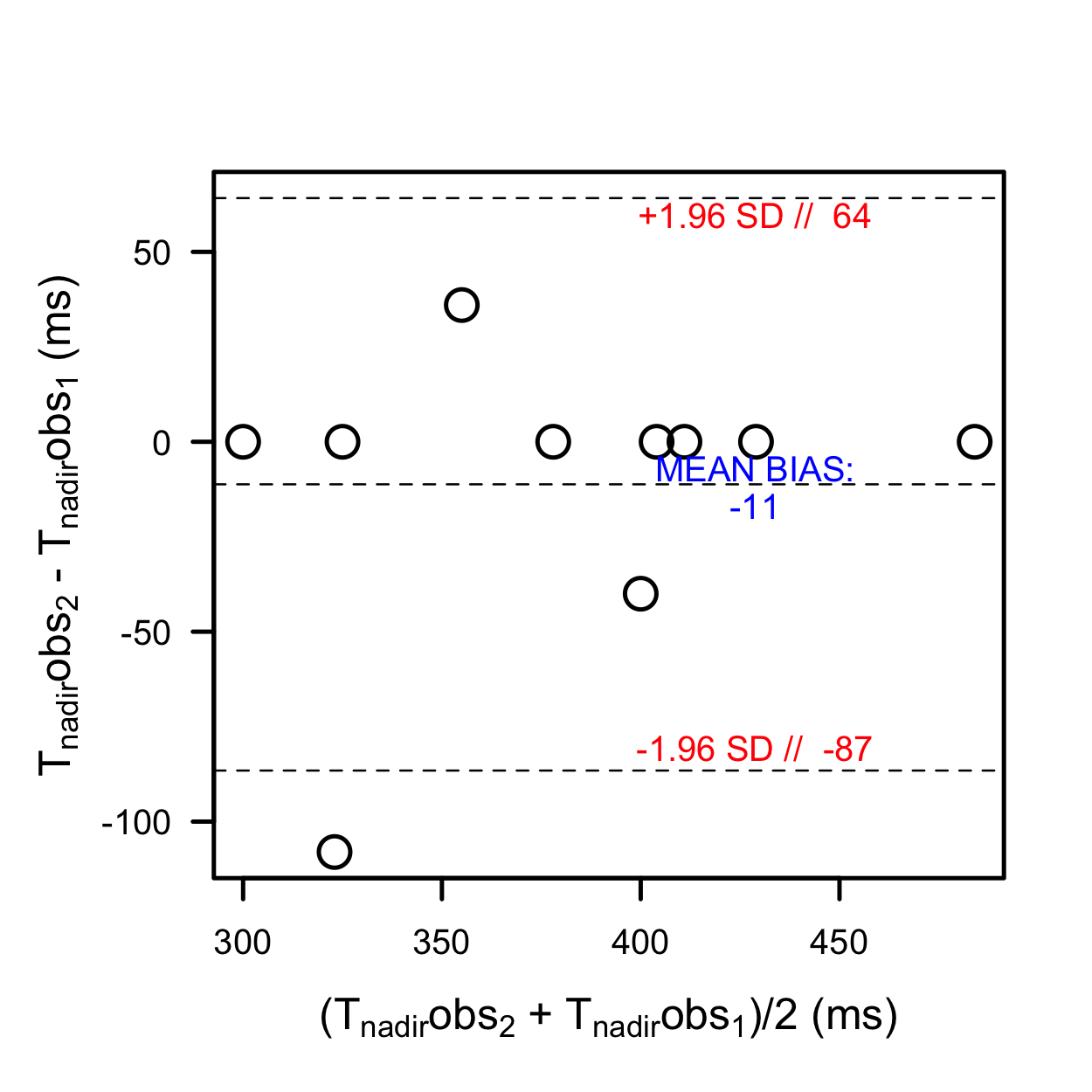 |
| 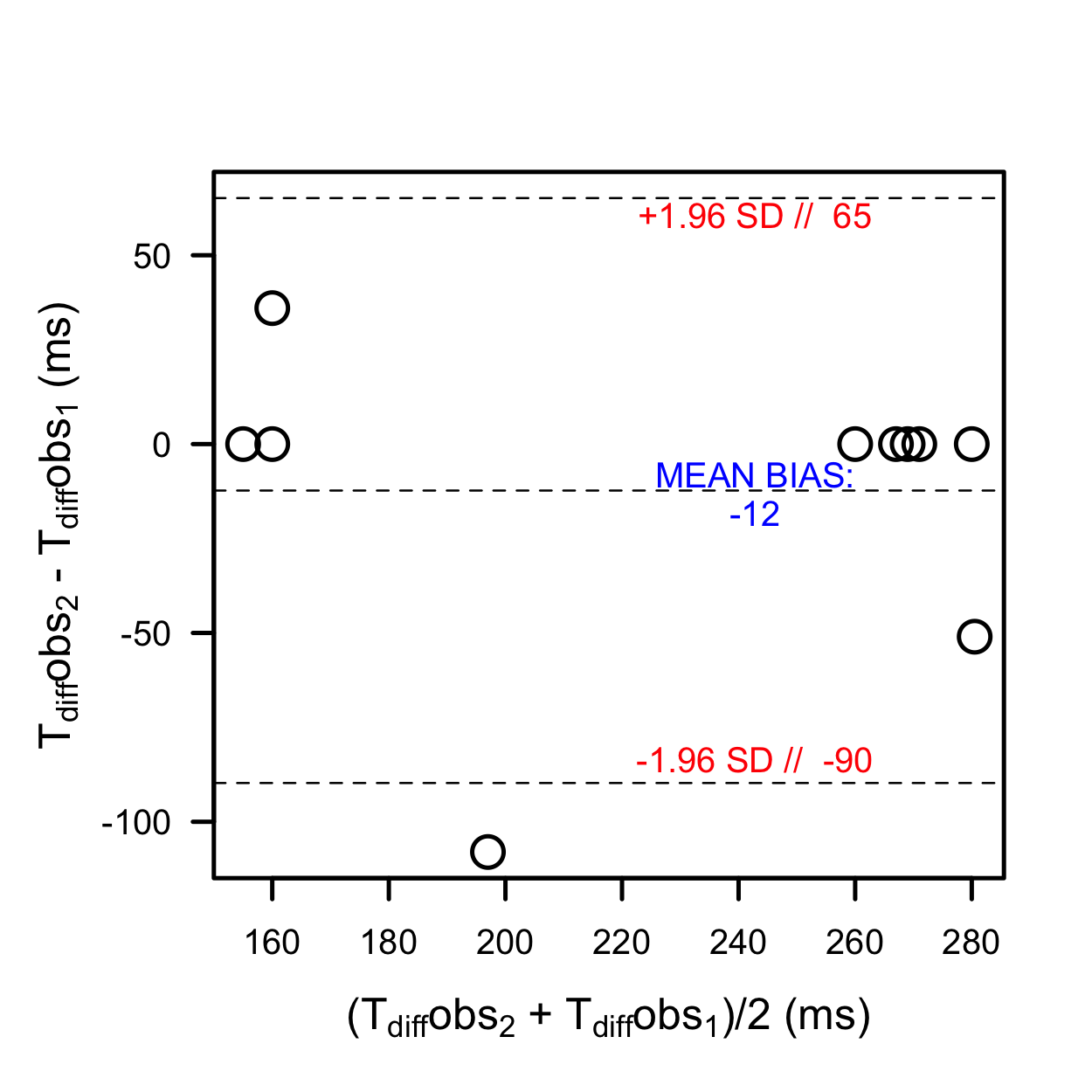 | 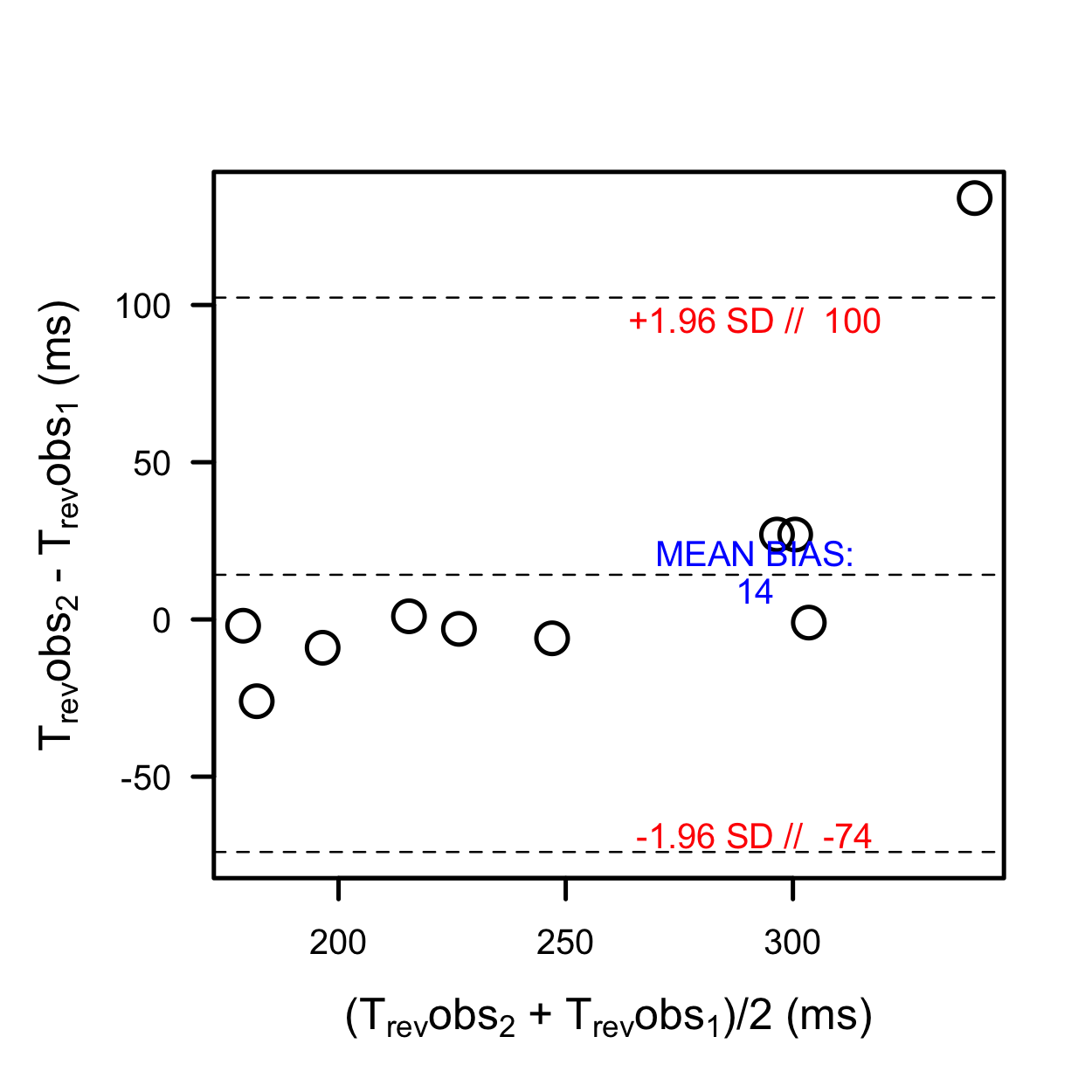 |
| 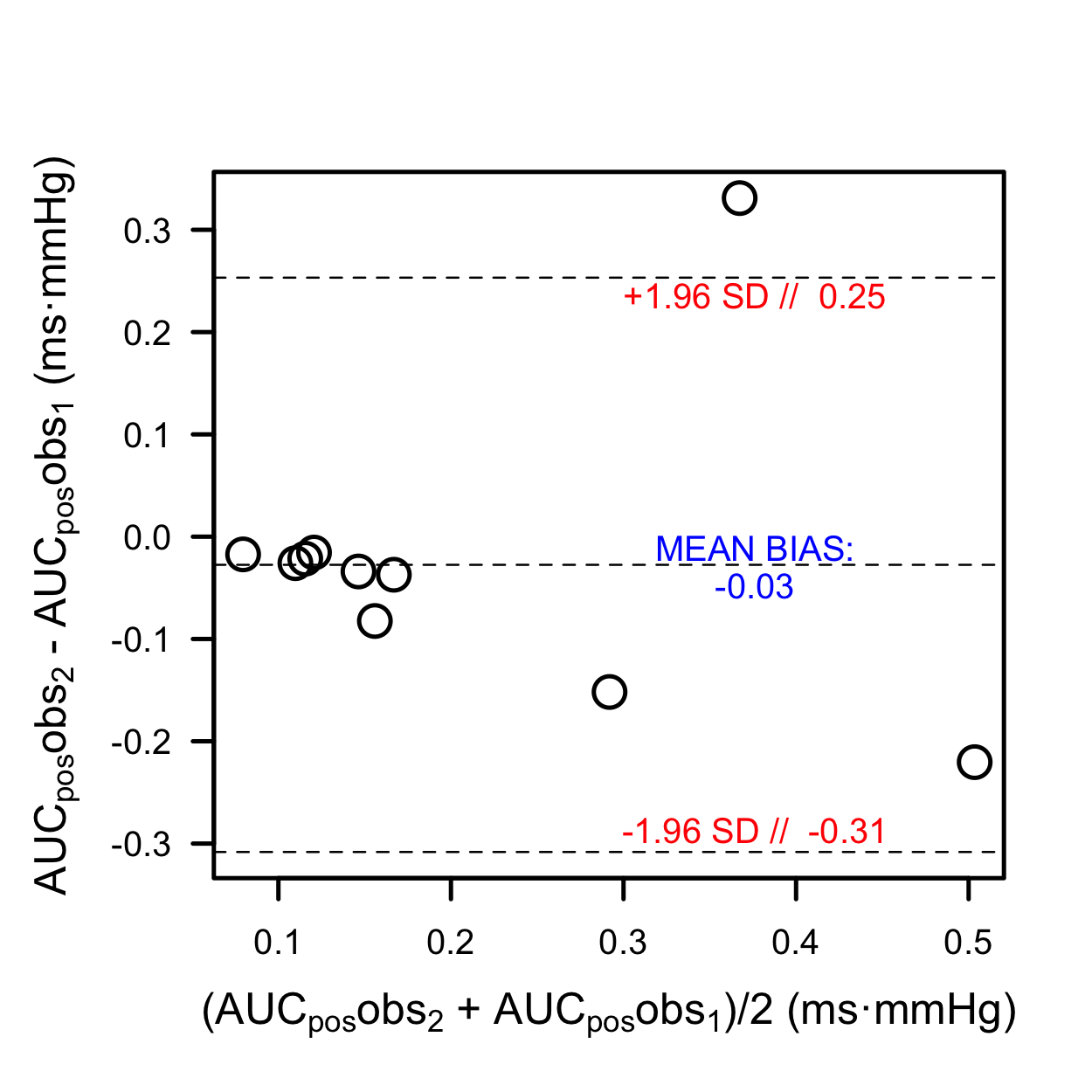 | 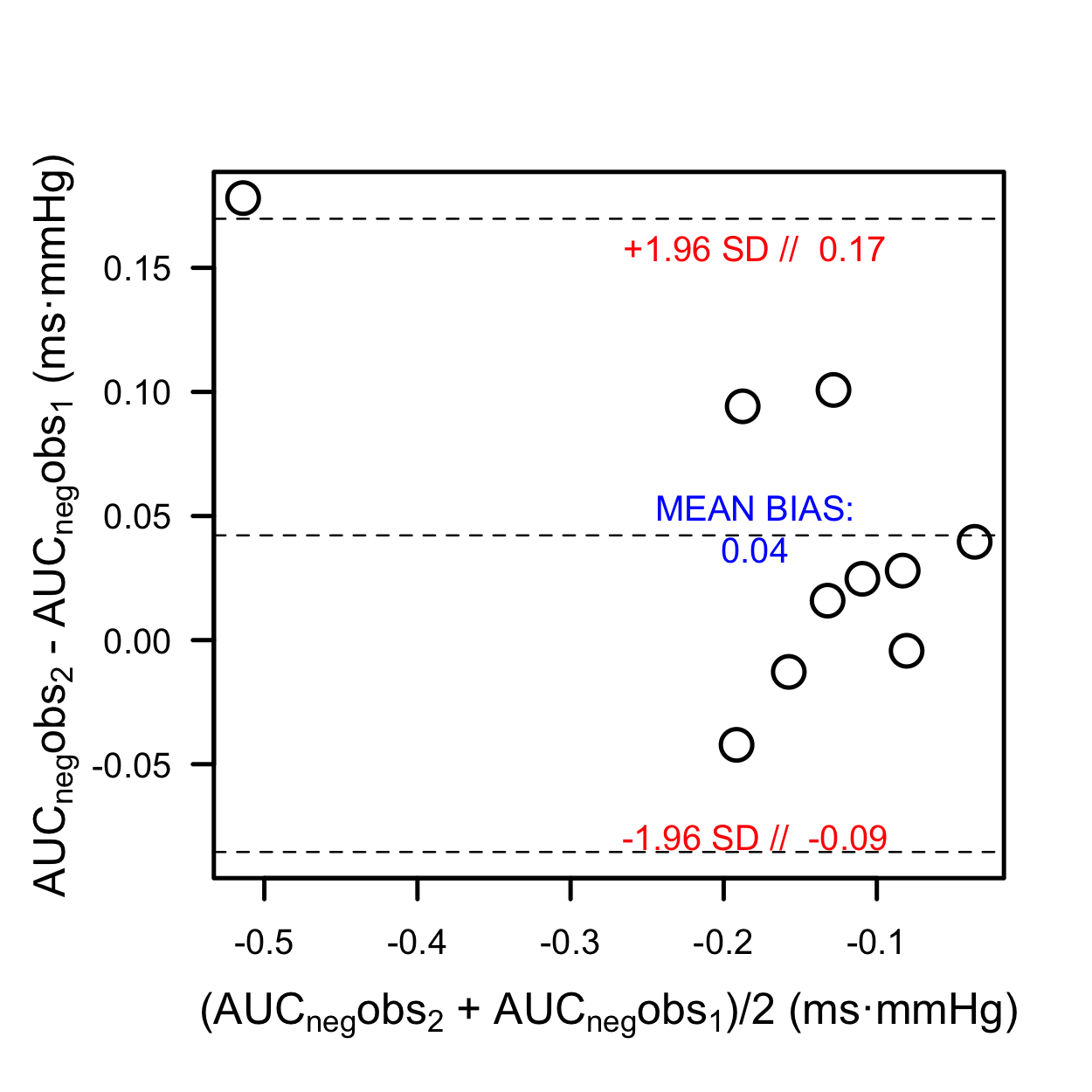 |

***Appendix 2, supplemental figure 2.****Intra-observer variability assessment using Bland-Altman plots which depict bias (mean difference, solid line) and 95% limits of agreement (dashed lines) for all variables derived from vWERP.*

*Abbreviations: AUC_neg_ - Area under the curve on the negative side of the vertical axis. AUC_pos_ – Area under the curve on the positive side of the vertical axis; Obs – observer; ∆P_nadir_ – nadir pressure gradient; ∆P_peak_ – peak pressure gradient*

| PVR ≤ 2 (n=10) | | |
| --- | --- | --- |
| 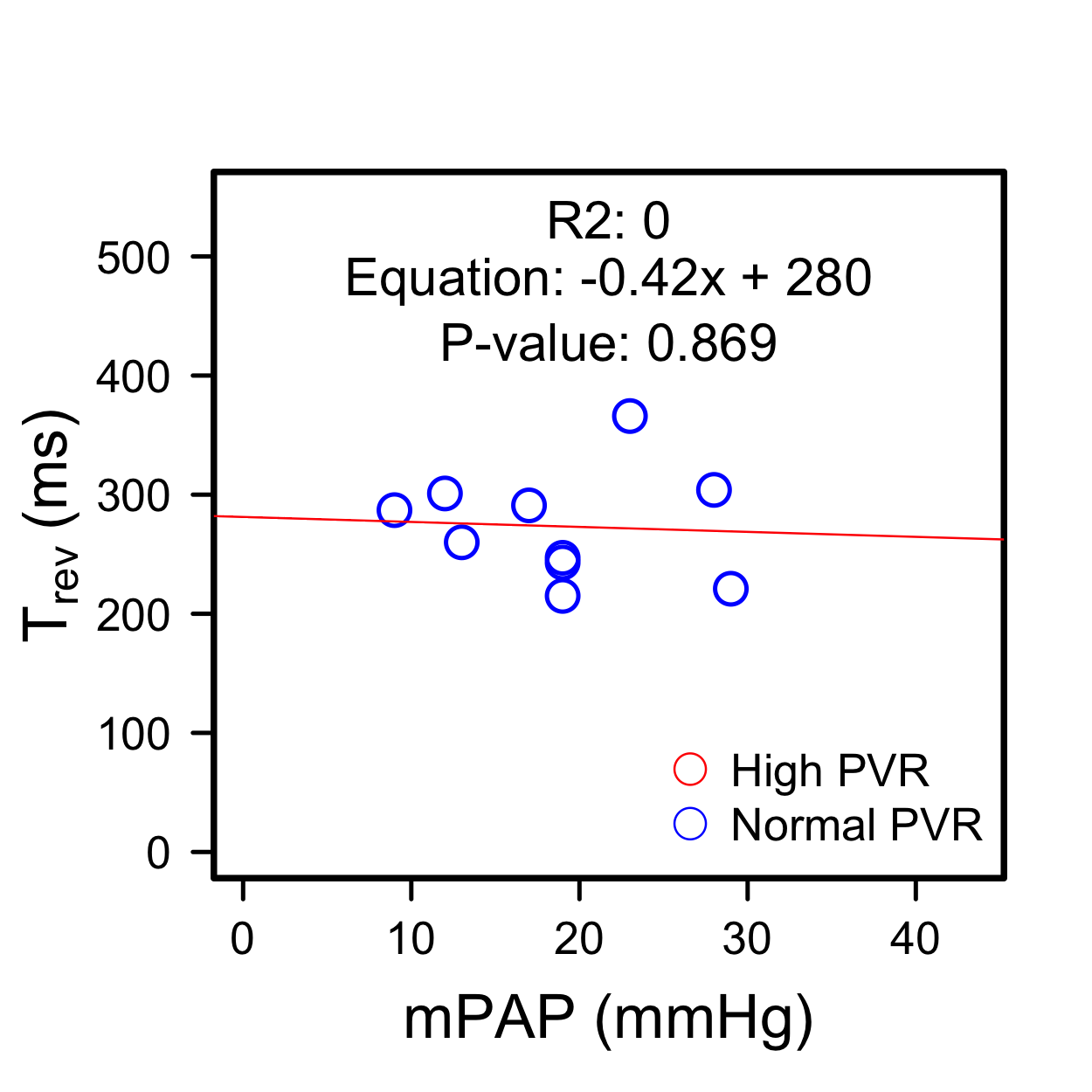 | 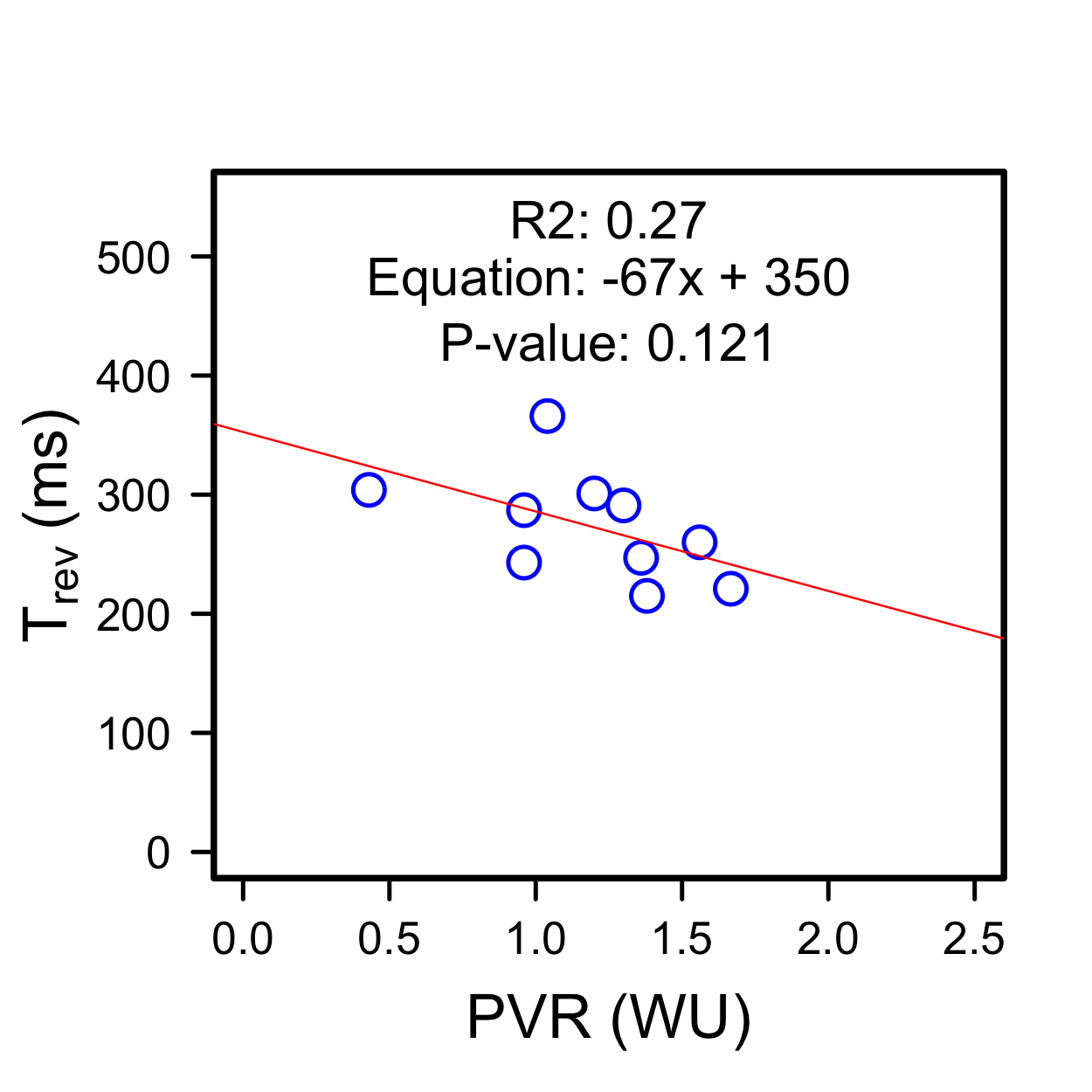 | 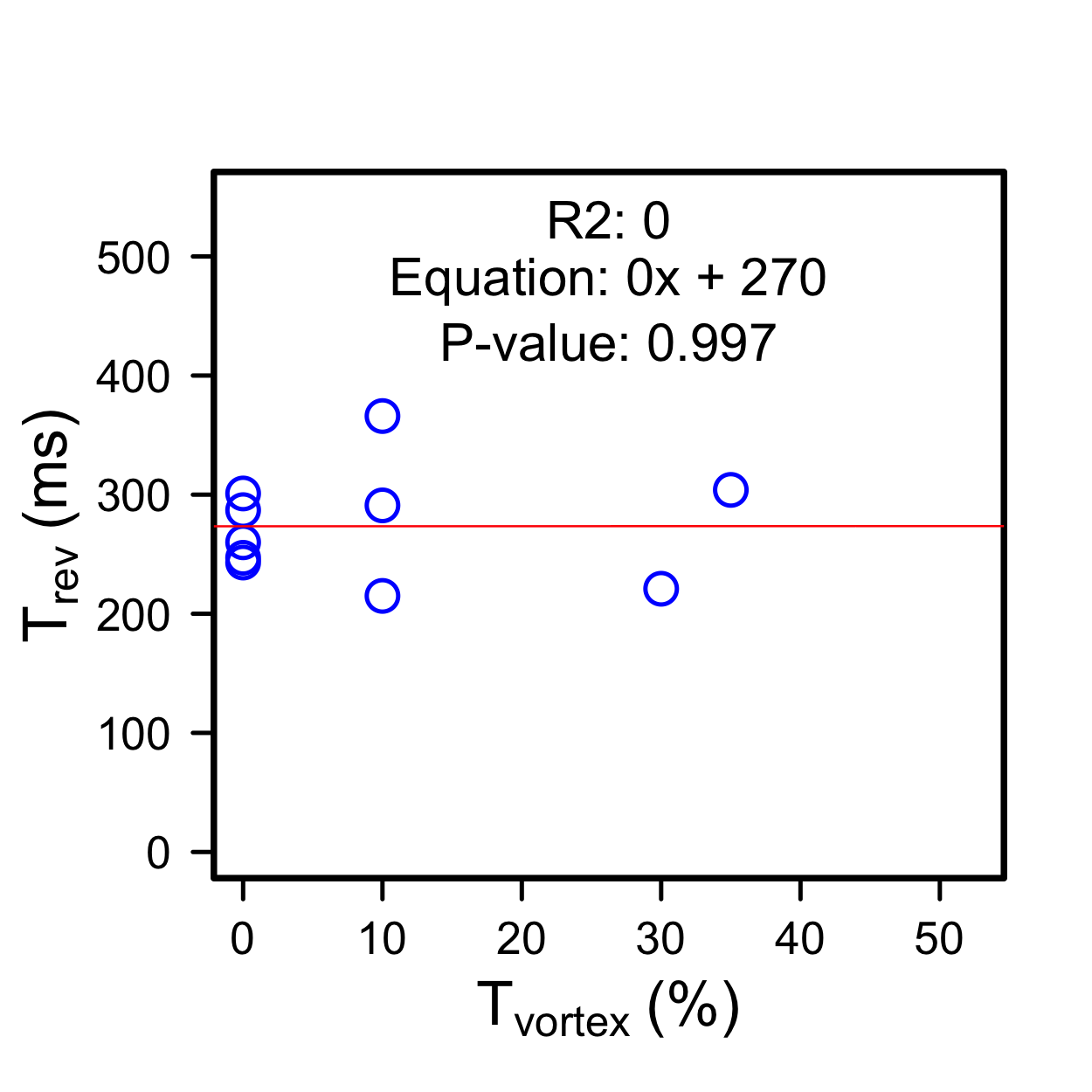 |
| PVR > 2 (n=26) | | |
| 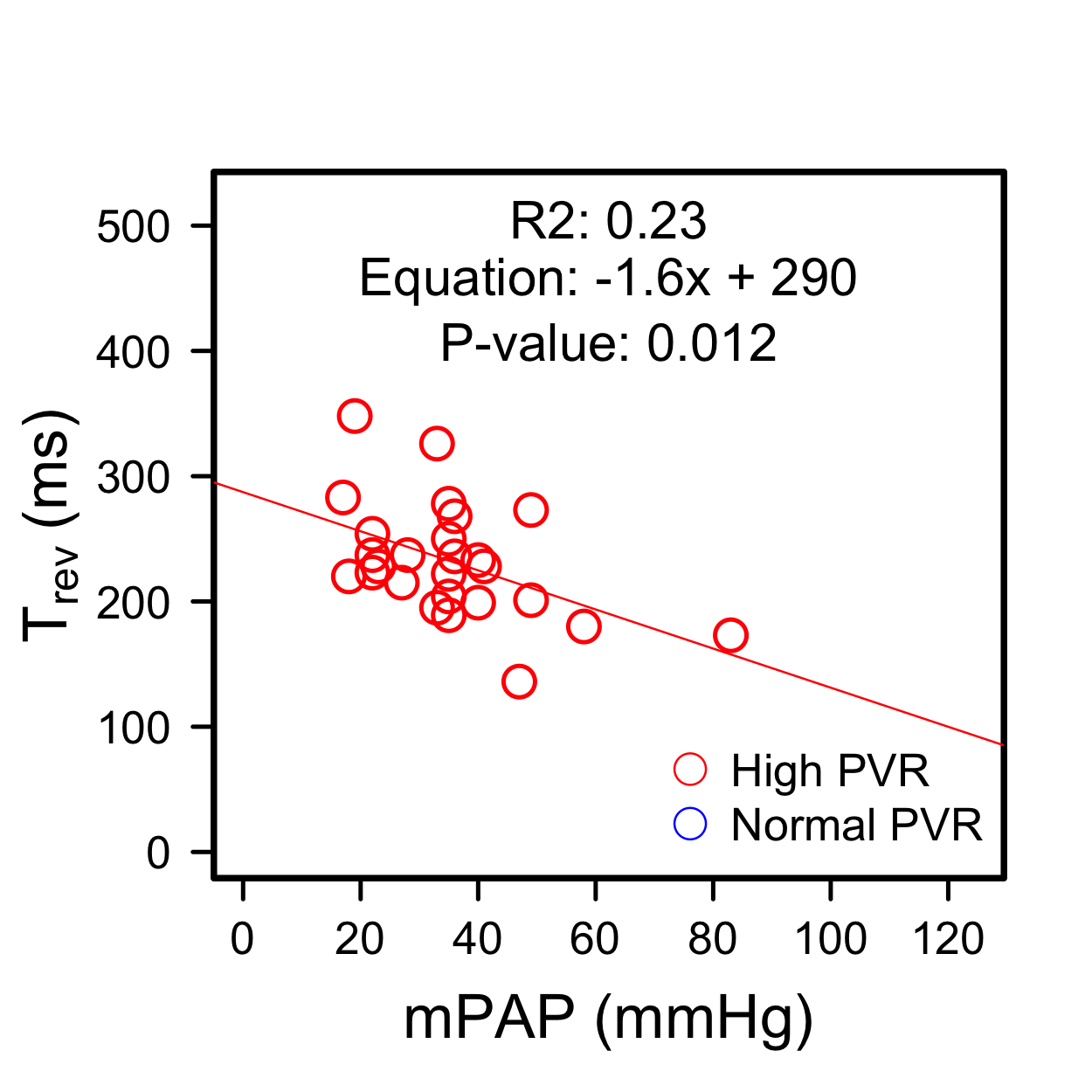 | 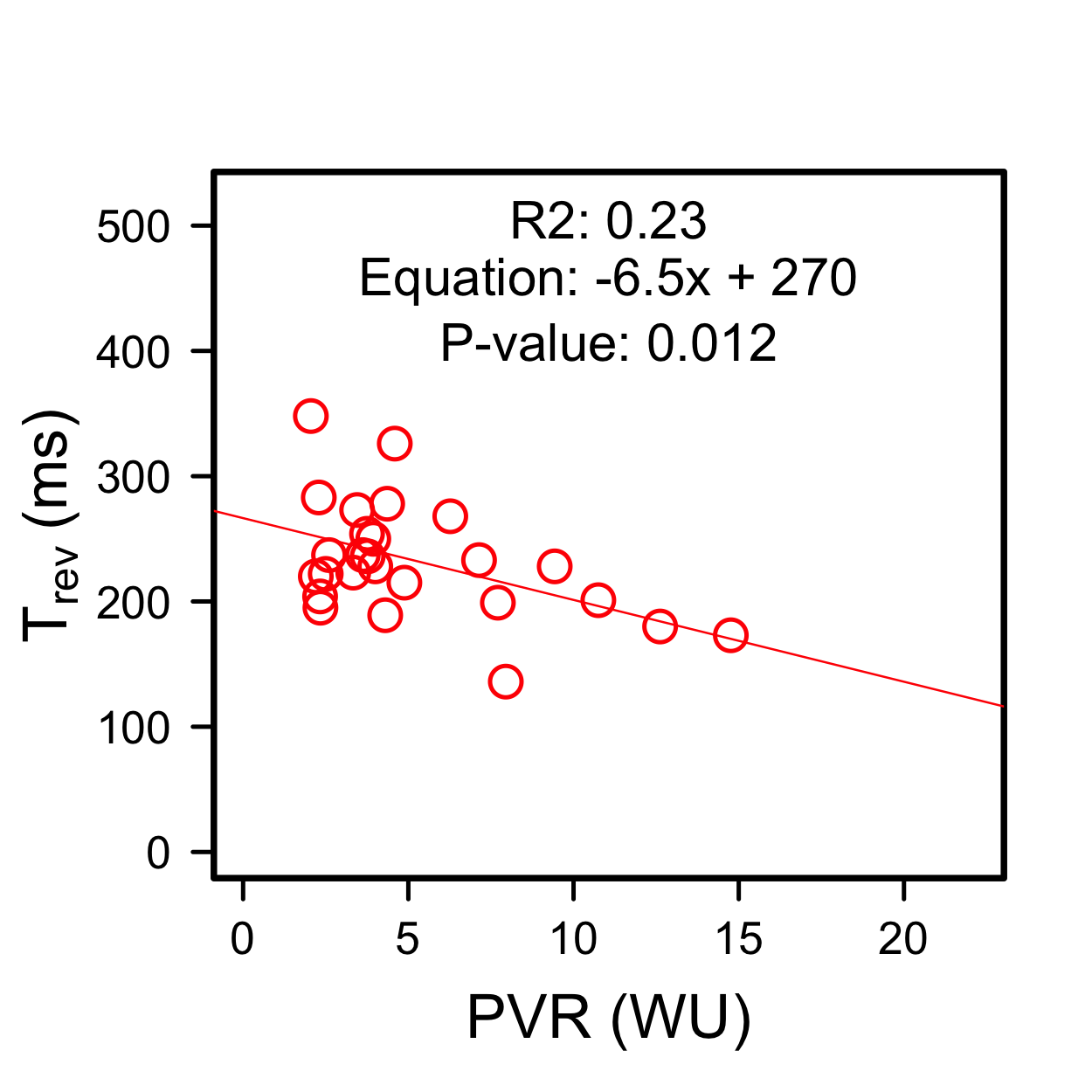 | 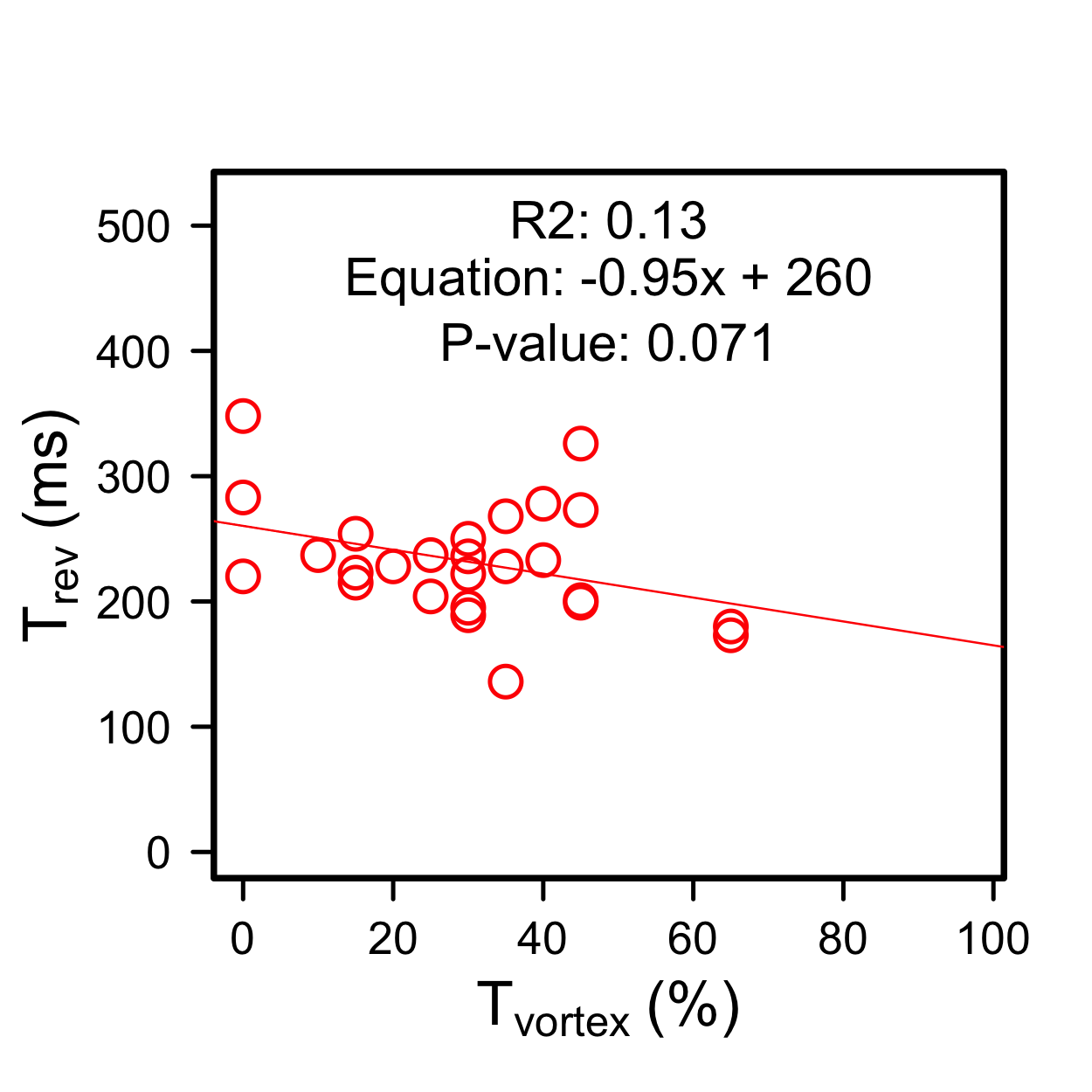 |

**Appendix 3 – subgroup analysis according to pulmonary vascular resistance**

***Appendix 3, supplemental figure 1.*** *Scatter plots of subgroups with the time to pressure gradient reversion (T_rev_) plotted against mean pulmonary artery pressure (mPAP) (left), against pulmonary vascular resistance (PVR) (middle), and against duration of vortical blood flow in the pulmonary artery (T_vortex_) (right). Results are presented in subgroups with normal PVR ≤ 2 WU and high PVR > 2 WU.*
